# Two TAL effectors of *Xanthomonas citri* promote pustule formation by directly repressing the expression of GRAS transcription factor in citrus

**DOI:** 10.1186/s43897-024-00131-1

**Published:** 2025-03-14

**Authors:** Yichao Yan, Xiaomei Tang, Zhongfeng Zhu, Ke Yin, Yikun Zhang, Zhengyin Xu, Qiang Xu, Lifang Zou, Gongyou Chen

**Affiliations:** 1 Shanghai Collaborative Innovation Center of Agri-Seeds/State Key Laboratory of Microbial Metabolism, School of Agriculture and Biology, Shanghai Jiao Tong University, Shanghai, 200240 China; 2 Shanghai Yangtze River Delta Eco-Environmental Change and Management Observation and Research Station, Ministry of Science and Technology, Ministry of Education, Shanghai, 200240 China; 3 National Key Laboratory for Germplasm Innovation and Utilization of Horticultural Crops, Huazhong Agricultural University, Wuhan, 430070 China; 4 Anhui Engineering Laboratory for Horticultural Crop Breeding, College of Horticulture, Anhui Agricultural University, Hefei, 230036 China

**Keywords:** *Xanthomonas citri*, Citrus, Canker, PthA, GRAS, PTG/Cas9

## Abstract

Citrus bacterial canker (CBC), caused by *Xanthomonas citri* subsp. *citri* (*Xcc*), poses a significant threat to the citrus industry. *Xcc* employs the transcription activator-like effector (TALE) PthA4 to target the major susceptibility (*S*) gene *CsLOB1* in citrus, promoting host susceptibility to bacterial canker. However, the contribution of other *Xcc* TALEs, aside from PthA4, to virulence remains underexplored. In this study, we characterized two PthA1 variants, designated PthA5 and PthA6, which facilitate *Xcc* infection in susceptible citrus species by promoting the formation of hypertrophy and hyperplasia symptoms. Both PthA5 and PthA6 bind directly to effector-binding elements (EBEs) in the promoter of *CsGRAS9*, suppressing its expression. *CsGRAS9* negatively regulates *Xcc* growth in citrus and contributes to CBC resistance. Notably, natural variations in the EBEs of the *FhGRAS9* promoter, a homolog of *CsGRAS9* in Hong Kong kumquat, prevent *Xcc* from affecting *FhGRAS9* expression. Using the PTG/Cas9 system, we generated *proCsGRAS9*-edited sweet orange lines #18–2 and #23, which contain 86-bp and 62-bp deletions in the EBE regions of the *CsGRAS9* promoter. These mutant lines showed enhanced *CsGRAS9* expression and increased resistance to CBC during *Xcc* infection. Several GA-related genes and *CsTAC1*, regulated by *CsGRAS9*, were also identified. This is the first report that TALEs act as repressors of a resistance gene to confer host susceptibility.

**Supplementary Information:**

The online version contains supplementary material available at 10.1186/s43897-024-00131-1.

## Core

The natural variations at EBE regions of *GRAS9s* promoter affect the binding affinity of TALE proteins (PthA4, PthA5, and PthA6), resulting in the differential expression of *GRAS9s*, which is induced in Hong Kong kumquat during *Xcc* infection. Mutations generated with 86-bp and 62-bp deletions in the EBEs of the *CsGRAS9* promoter by the PTG/Cas9 system resulted in an increased canker resistance in sweet oranges, providing a potential resource for the disease-resistant genetic breeding of cultivated citrus.

### Gene and accession numbers

Sequences were obtained from the National Center for Biotechnology Information database (NCBI), Arabidopsis Information Resource (TAIR) (https://www.arabidopsis.org/), Rice Genome Annotation Project (http://rice.uga.edu/), Sol Genomics Network (https://solgenomics.net/), and Citrus Pan-genome to Breeding Database (CPBD) (http://citrus.hzau.edu.cn/index.php). The accession numbers are listed in Supplementary Table S2 and Table S3.

## Introduction

Citrus bacterial canker (CBC), caused by *Xanthomonas citri* subsp. *citri* (*Xcc*), is a severe disease that significantly affects citrus production and results in substantial economic losses (Stover et al. 2014). *Xcc* strains (pathotype A) have an extensive geographic distribution and infect various hosts. Citrus has a diverse collection of germplasm resources, including grapefruit (*Citrus paradisi*), sweet orange (*Citrus sinensis*), lemon (*Citrus limon*), and pummelo (*Citrus grandis*), all of which are susceptible to citrus canker (Duan et al. 2022). In addition, the *Xcc* pathotypes A* and A^w^ have a more limited host range and infect alemow (*Citrus macrophylla*) and Mexican lime (*Citrus aurantifolia*), but not grapefruit (*Citrus paradisi*) (Patané et al. 2019). Ongoing efforts to breed profitable citrus cultivars that are resistant to disease remain a crucial task. During infection, bacteria compress intercellular spaces, leading to cell hydration and swelling (An et al. 2020). *Xanthomonas* species promote xanthan gum synthesis, which enhances water absorption through capillary action in the xylem (Shahbaz et al. 2022). Canker symptoms first appear as water-soaked lesions on the abaxial surface of leaves, which then develop into eruptive lesions caused by rupture and splitting of the outer leaf layer, ultimately leading to leaf loss. Kumquats (*Fortunella* spp.) exhibit resistance to *Xcc* through early leaf abscission and development of a hypersensitive response (HR)-like phenotype (Teper et al. 2020). Swelling, division, and proliferation of host cells after infection contribute to the water-soaked appearance (Brunings et al. 2003, Zou et al. 2021). Further research is needed to clarify the ongoing arms race between plant defense mechanisms and pathogens striving to evade or overcome these defenses.

*Xanthomonas* spp. secrete transcription activator-like effectors (TALEs) into plant cells through the type III secretion system (T3SS) to manipulate bacterial virulence in hosts (Ji et al. 2016). TALE proteins are potent and essential factors that directly bind to specific DNA bases in promoters through interactions with the 12th and 13th repeat variable diresidues (RVDs) located in the central region of polymorphic repeats (CRRs) (White et al. 2009). The virulence of *Xcc*A strains mainly depends on the interaction between PthA4 and the effector binding element (EBE) region in the promoter of the canker susceptibility (*S*) gene *CsLOB1* (Hu et al. 2014, Li et al. 2014). In plant–pathogen interactions, TALEs reprogram host cells to promote hypertrophy and hyperplasia, leading to morphological and physiological changes that enhance favor pathogen fitness, proliferation, and spread, or trigger disease resistance (Timilsina et al. 2020). The activation of *CsLOB1* expression is essential for pustule formation, cell wall modifications, and cell expansion (Zou et al. 2021, Li et al. 2014). *Xcc*A strains also contain three additional homologs of PthA: PthA1, PthA2, and PthA3 (Yan et al. 2012). In the citrus variety ‘Pera’, PthA1 induces the expression of the *S* gene *DIOX*, promoting the formation of necrotic pustules and facilitating pathogen adaptation (Abe et al. 2016). However, the precise mechanisms through which PthA1 and its derivatives promote necrotic pustule formation and pathogen adaptability in hosts remain unclear and ambiguous. *X*. *citri* strains use horizontal gene transfer, which is mediated by plasmid diversity arising from human activities and plant transportation, to circumvent host resistance and adapt to environmental changes (Pruvost et al. 2021). TALE variations arise through recombination, replication, insertions/deletions (InDels), and single nucleotide polymorphisms (SNPs) in the CRR region, driving a co-evolutionary arms race between pathogens and host resistance (Teper et al. 2021, Ruh et al. 2017, Ferreira et al. 2015). In response to pathogen infection, plants trigger a type of programmed cell death (PCD) known as HR. The short PthA4^AT^ from *X*. *citri* A^T^ strains, which contains 7.5 repeats in the RVD region, elicits HR and resistance in *C*. *limon* and *C*. *sinensis*, suggesting that its function extends beyond “classical” TALEs (Roeschlin et al. 2019). Another TALE, Tal2_Xss-V2-18_, with 25.5 repeats in the RVD region, is a critical component for the full virulence of *X*. *citri* pv. *malvacearum* (*Xcm*) strains in cotton (Haq et al. 2020). In our previous study, we isolated two highly pathogenic strains, Xcc003 and Xcc086, from infected grapefruit and kumquat leaves, respectively (Ye et al. 2013). However, the contribution of the long *avrBs3*/*pthA* variants in these strains to bacterial virulence remains unknown.

Repetitive regions of TAL effectors specifically target EBEs in gene promoters to regulate gene expression and facilitate disease progression. Xu et al. (2019) used the CRISPR system to generate broad-spectrum resistance in rice by introducing gene-edited mutations in the EBE regions of the *OsSWEET13* promoter (Xu et al. 2019). Similarly, mutations in the EBE_PthA4_ regions of the *CsLOB1* promoter in citrus plants reduced canker symptoms by using polycistronic tRNA–gRNA/Cas9 (PTG/Cas9) and Cas12a-mediated genome editing systems (Su et al. 2023; Tang et al. 2021a). However, the relationship between EBE mutations in target promoters and citrus resistance remains insufficiently understood, especially in citrus plants. Primitive and wild citrus germplasms suppress the development of canker symptoms following *Xcc* infection, supporting the use of natural genetic resources in breeding for improved canker resistance (Fu et al. 2020). Recently, Tang et al. (2021b) reported natural variation in the *AbLOB1* promoter in *Atalantia buxifolia* (Chinese box orange, a primitive Chinese variety) and demonstrated that mutations in the *AbLOB1* promoter led to distinct *LOB1* transcription levels compared with *C. sinensis*, altering citrus canker resistance (Tang et al. 2021b). Thus, both natural variations and genome editing targeting EBE regions have been used to generate broad-spectrum resistant plants (Su et al. 2023). However, natural variation in promoters and similar disease resistance mechanisms in resistant Hong Kong kumquat (*Fortunella hindsii*) have not been reported. The susceptibility or resistance genes associated with citrus canker in Hong Kong kumquat remain to be validated, and the target gene directly regulated by TALEs is yet to be identified.

GRAS proteins are plant-specific transcription factors that play a pivotal role in regulating stress response, gibberellin (GA) signaling, and plant development (Hirsch et al. 2009). In *Arabidopsis*, *miR171c* targets GRAS proteins to regulate various biological processes, including the branching of the shoot and maintenance of the shoot meristem (Wang et al. 2010). In addition, SCARECROW-LIKE (SCL) proteins function pleiotropically in rice growth and disease resistance. After the inoculation of rice by *Magnaporthe oryzae*, the expression and transcriptional activity of *OsSCL7* were increased, resulting in the elevated expression of defense-related genes and enhanced rice resistance to *M*. *oryzae* (Lu et al. 2022). *miR171b* inhibits the transcription of *SCL6-IIs* to enhance defense responses against *M*. *oryzae* and increase resistance to rice blast (Li et al. 2022a, b). Moreover, *CMLs* and *GRAS*s contribute to disease resistance in sesame genotypes resistant to *Macrophomina phaseolina* (Yan et al. 2021). *CsGRAS9* (*Cs2g22130*) has been classified into the AtSHR subfamily (Zhang et al. 2019). Our previous study demonstrated that the loss of function of TAL effectors induced *Cs2g22130* expression during *Xcc* infection (unpublished data). Recently, Teper et al. (2020) reported that PthA4 downregulates the expression of *CsGRAS9* at 4 days post infection of *Xcc*. These findings suggest that *CsGRAS9* is involved in the response to *Xcc* infection and could serve as a potential target for TAL effectors. However, the characterization of *CsGRAS9* function in response to biotic stress and the role of GRAS9s proteins in citrus species with varying levels of canker resistance have not been adequately explored. Plant responses to biotic stress involve regulation of phytohormone levels, telomerase activity, and senescence (Kalinina et al. 2018). For example, the suppression of the *AtSCL32* homolog *SlGRAS26* in tomato impairs GA biosynthesis by downregulating the expression of the GA metabolism genes *GA3ox1* and *GA3ox2* and the GA biosynthesis genes *CPS* and *KAO* while activating the GA inactivation pathway (Zhou et al. 2018). IDD proteins function as transcriptional coactivators with the DELLA protein REPRESSOR OF GA1-3 (RGA) to promote the expression of *SCL3* and regulate the GA signaling pathway (Jaiswal et al. 2022). Plants have evolved mechanisms to maintain telomere length by regulating telomerase activity, thereby preventing chromosome degradation, senescence, and eventual cell death due to telomere shortening (Procházková Schrumpfová et al. 2016). The telomerase activator *TAC1* may play a role in plant defense mechanisms in olive trees (*Olea europaea* L. subsp. *europaea* var. *europaea*), which affects auxin signaling (Ramírez-Tejero et al. 2021). In highly resistant cultivars, *TAC1* expression is suppressed in roots, whereas in extremely susceptible cultivars, its expression is induced. *Cs5g06630/TAC1* is orthologous to *Arabidopsis thaliana TAC1*, which enhances auxin responses and induces telomerase activity in plant leaves (Ren et al. 2007). However, the downstream mechanisms regulated by *CsGRAS9* that affect citrus canker susceptibility remain unclear.

The present study examined the mechanism of TALEs action in pathogen dispersal and pathological hypertrophy in citrus hosts to breed varieties resistant to *Xcc* infection. We characterized two *avrBs3/pthA* variants, *pthA5* and *pthA6*, which promote bacterial pathogenesis and symptom development in hosts. *CsGRAS9* expression was suppressed in response to PthA4, PthA5, and PthA6 during *Xcc* infection. We found that PthA5 and PthA6 directly bind to EBEs in the *CsGRAS9* promoter, thereby suppressing gene expression in grapefruit. We also identified the transcription factor *FhGRAS9* in the resistant *F. hindsii* ‘Hong Kong kumquat’. In Hong Kong kumquat, in addition to the *FhGRAS9* promoter genotype that aligns with the susceptible grapefruit *CsGRAS9* promoter (named *FhGRAS9*-P1), we detected a natural variation (a 558-bp deletion) in the *FhGRAS9* promoter (named *FhGRAS9*-P2). Moreover, we determined that natural variations in the promoters of *GRAS9s* lead to differential gene expression in the two citrus germplasms, which exhibited contrasting levels of canker resistance. Overexpression of *CsGRAS9* conferred tolerance to citrus canker in grapefruit. We also generated *CsGRAS9* promoter-edited *C. sinensis* cv. ‘Anliu’ lines using the PTG/Cas9 system, demonstrating that editing the PthA4, PthA5, and PthA6 EBEs in the *CsGRAS9* promoter region enhanced resistance to *Xcc* in sweet orange. These results elucidate the molecular mechanism of *CsGRAS9* in citrus plants during *Xcc* infection, providing a potential resource for the disease-resistant genetic breeding of cultivated citrus.

## Results

### PthA1 variants PthA5 and PthA6 promote the canker symptom formation

**Fig. 1:**
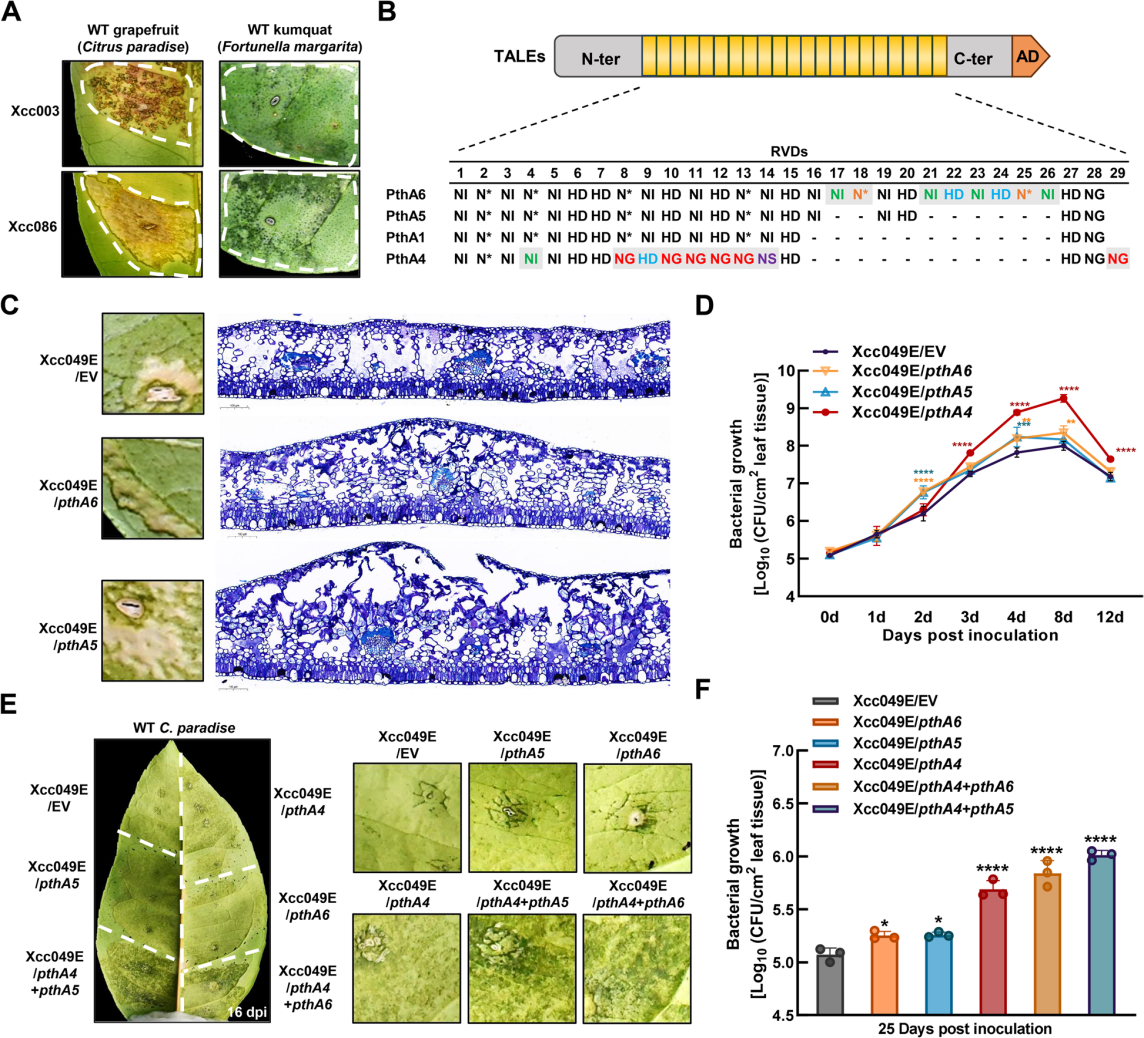
Effect of PthA5 and PthA6 in canker formation. **A** Grapefruit (*Citrus paradisi*) and kumquat (*Fortunella margarita*) leaves were infiltrated with Xcc003 and Xcc086 strains (OD_600_ = 1.0). Canker symptoms were recorded at 21 days post-inoculation (dpi). **B** Alignment of repeat-variable diresidues (RVDs) of PthA6, PthA5, PthA1, and PthA4. PthA6 (from Xcc003) and PthA5 (from Xcc086) were compared with PthA1 and PthA4 from *X*. *citri* strains. Black lines (-) indicate missing RVDs. Different RVDs are highlighted in gray and colored, corresponding to nucleotide association and DNA base specificities. Green: NI recognizes adenine (A); Red: NG recognizes thymine (T); Blue: HD recognizes cytosine (C); Orange: N* recognizes C and T; Purple: NS recognizes ACT and guanine (G). **C** Grapefruit leaves were infiltrated with *Xcc* suspensions (OD_600_ = 1.0) of *tal*-free deletion mutant Xcc049E/EV and complemented strains Xcc049E/*pthA5* or Xcc049E/*pthA6*. Canker symptoms were evaluated at 21 dpi. Microscopic observation of thin cross-sections in the circled regions. **D** Effect of PthA5 and PthA6 on bacterial growth in grapefruit. Grapefruit leaves were infiltrated with *Xcc* suspensions (OD_600_ = 0.8). Bacterial growth assays of *Xcc* strains were monitored at 0, 1, 2, 3, 4, 8, and 12 dpi. Bacterial growth is presented in mean colony-forming units (CFU)/cm^2^, indicating PthA5 and PthA6 enhance bacterial growth in citrus host. Error bars represented standard deviations of three biological replicates. Asterisks denote statistically significant differences in bacterial-inoculated leaves, as determined by two-way ANOVA with Dunnett’s test: **p < 0.01, ***p < 0.001, and ****p < 0.0001. **E** Grapefruit leaves were infiltrated with *Xcc* suspensions (OD_600_ = 0.6) of Xcc049E/*pthA5*, Xcc049E/*pthA6*, and mixed suspensions of Xcc049E/*pthA4* and Xcc049E/*pthA5* or Xcc049E/*pthA*6 at a 1:1 ratio. Xcc049E/EV and Xcc049E/*pthA4* were used as the negative and positive controls, respectively. Citrus canker symptoms were evaluated at 16 dpi. **F** Bacterial growth of *Xcc* strains in grapefruit leaves was monitored at 25 dpi. Error bars represent standard deviation of three biological replicates. Asterisks denote statistically significant differences, as determined by one-way ANOVA with Dunnett’s test: *p < 0.05, ****p < 0.0001

Our previous report highlighted significant variability in the size of the *tal* genes from different Chinese *Xcc* strains (Ye et al. 2013). Among these, strains Xcc086 and Xcc003 were shown to contain unique *tal* genes that differ from the *pthA4* gene and *tal* genes found in other Chinese *Xcc* strains (Ye et al. 2013). Given the mode of action of TALE proteins, these variants are expected to play specific roles during bacterial pathogenesis. The pathogenicity of Xcc003 and Xcc086 was confirmed in susceptible grapefruit ‘Paradise’ and resistant kumquat (Fig. 1A). To define the unique *tal* genes of Xcc086 and Xcc003, we cloned a 3.6-kb band from Xcc086 (corresponding to the second hybridization signal band), and a 4.4-kb band from Xcc003 (corresponding to the first hybridization signal band), from the *BamH*I-digested plasmid DNA of Xcc086 and Xcc003 according to our previous Southern blot assays (Ye et al. 2013). The *BamH*I bands, containing the central RVD repetitions, were sequenced (Supplementary Fig. S1). The 3.6-kb band of Xcc086 contains a *tal* gene encoding a TALE with 19.5 RVD repeats, and the 4.4-kb *BamH*I band of Xcc003 corresponds to a *tal* gene encoding a TALE with 27.5 RVD repeats (Fig. 1B and Supplementary Fig. S1). We name these TALE proteins PthA5 and PthA6, respectively. Alignment analysis of the RVDs revealed that the region of PthA5 from the 1st to the 15th and the last two repeats was identical to all the repeats of PthA1 (Fig. 1B). The regions of PthA6 resembled those of PthA5 and PthA1, but contained 8 and 11 additional RVDs compared to PthA5 and PthA1, respectively (Fig. 1B). Notably, the 21nd to 26th extra RVDs of PthA6 matched the 9th to 14th central repeats of PthA5 and PthA1, based on the arrangement of the RVD repeats (Fig. 1B). These analyses indicate that PthA5 and PthA6 belong to the members of the PthA1 group. Additionally, repeat-based phylogenetic lineages of PthA5, PthA6, and 262 TALEs obtained from 77 fully sequenced *X*. *citri* strains in the NCBI database were assessed using DisTAL. On the phylogenetic tree, PthA5 and PthA6 clustered together with PthA1, PthAW1, Apl3, TAL4_Xc03-1638-1-1_, and TALEs from *Xcc* and *Xcm* strains isolated from diverse geographic regions, suggesting the common ancestral origin (Supplementary Fig. S2A and S2C). TAL4_Xc03-1638-1-1_ was identified in *X. citri* strain Xc-03–1638-1-1 and was conserved among other *X. citri* strains, including LJ207–7, LL074–4, LH276, and LH201. PthA5 also clustered with TAL1 of *Xcc* LJ207-7 strain (from the Southwest Indian Ocean islands), which is encoded by a plasmid and is closely related to TAL1 of TX160197 in the PthAW1 and AW pathotypes (Supplementary Fig. S2A). Interestingly, PthA6 was most closely related to TAL1 of the Korean strain *X*. *citri* pv. *glycines* (*Xcg*) 1018, which is encoded on the chromosome. As TALE genes are genetically unstable, the adaptability of *Xanthomonas* species is enhanced by recombination events and RVD polymorphisms that arise under selective pressure during evolution (Schandry et al. 2018). Based on the clustering in repeat-based phylogeny, we speculate that recombination events likely occurred among ancestral PthA homologs from *Xcc*, *Xcg* and *Xcm* strains, leading to the variations in repeat numbers seen in PthA5 and PthA6.

To assess whether PthA5 and PthA6 play roles in *Xcc* pathogenicity, we constructed two pHZY derivative plasmids expressing PthA5 and PthA6, and introduced these recombinant plasmids into Xcc049E*,* a mutant strain of Xcc049 with deletions of all *tal* genes, generating Xcc049E/*pthA5* and Xcc049E/*pthA6*. The expression of PthA5 and PthA6 in the transformants was confirmed by western blotting (Supplementary Fig. S3). Bacterial titers of Xcc049E/*pthA5* and Xcc049E/*pthA6* were inoculated into grapefruit via infiltration to evaluate bacterial virulence and pathogenicity. Both Xcc049E/*pthA5* and Xcc049E/*pthA6* strains complemented Xcc049E's function to cause typical canker symptoms including hyperplasia and hypertrophy (Fig. 1C). The removal of TALEs effectively eliminated citrus cankers in grapefruit, and we observed that PthA5 and PthA6 positively contributed to disease symptom development. Microscopic observations further confirmed that PthA5 and PthA6 promoted canker symptom formation. Compared to leaves infected with Xcc049E/EV, the spongy parenchyma cells in grapefruit leaves infected with Xcc049E/*pthA5* and Xcc049E/*pthA6* were significantly enlarged (2–3 times), and the palisade cells infected with Xcc049E/*pthA5* were wider and more cratered at 21 days post-inoculation (dpi)(Fig. 1C). Furthermore, bacterial growth of Xcc049E/*pthA5* and Xcc049E/*pthA6* was significantly higher than Xcc049E at 2 and 4 dpi, though slightly lower than Xcc049E/*pthA4* (Fig. 1D). These results indicate that PthA5 and PthA6 function as virulence factors, inducing canker symptom formation in hosts.

Both Xcc003 and Xcc086 strains have the capacity to induce *CsLOB1* transcriptional activity due to the presence of *pthA4* (Ye et al. 2013) . In contrast, after inoculation with Xcc049E strains containing *pthA5* and *pthA6*, no significant differences in *CsLOB1* expression were observed compared to Xcc049E alone (Supplementary Fig. S4). To further investigate the molecular mechanisms of PthA5 and PthA6, we used FuncTAL for functional analysis based on DNA-binding affinity. The analysis revealed that PthA5 and PthA6 clustered with PthA1 (Supplementary Fig. S2B)**.** As shown in Supplementary Fig. S5, *CsLOB2* and *CsDIOX* were induced in response to PthA5, consistent with previous findings that PthA1 enhances the expression of *CsLOB2* and *CsDIOX* (Abe et al. 2016). TALgetter also predicted that the binding sites of PthA1 and PthA5 overlap in the promoter of *CsDIOX*, reinforcing the hypothesis that PthA1 and PthA5 activate transcription in the host by binding to the EBE site (Supplementary Fig. S6). Compared to individual infection with Xcc049E/*pthA4*, the simultaneous presence of *pthA4* and either *pthA5* or *pthA6* elicited more severe water-soaked canker symptoms and promoted bacterial growth in grapefruit (Fig. 1E-F). In agreement with previous observations, PthA5 functions similarly to PthA1, playing an additive role in PthA4-elicited pustule formation and acting as a pathogenicity factor by activating *DIOX* expression.

### PthA5 and PthA6 directly bind to the *CsGRAS9* promoter to inhibit its transcription

**Fig. 2:**
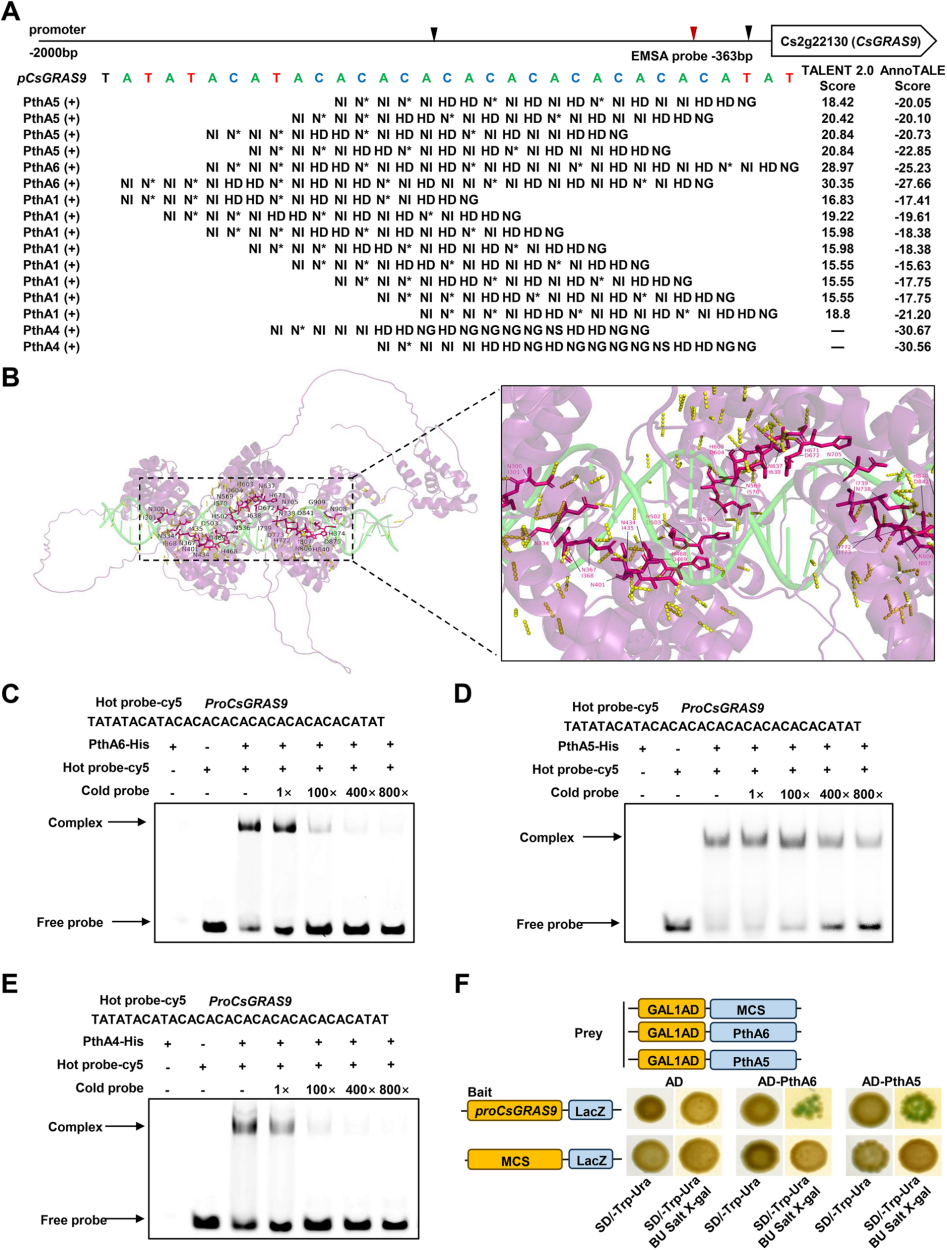
PthA4, PthA5, and PthA6 directly bind to the promoter of *CsGRAS9*. **A** Composition of the effector binding elements (EBEs) in the *CsGRAS9* promoter. Specific nucleotides in the predicted EBEs that match individual RVD sequences of PthA5, PthA6, PthA1 and PthA4 from *X*. *citri* are shown above. AnnoTALE’s "Predict and Intersect Targets" tool, incorporating TALENT 2.0, was used to efficiently scan the host promoterome and identify target EBE regions for TALEs. Comprehensive analysis of the putative target EBEs in the *CsGRAS9* promoter for PthA1, PthA4, PthA5, and PthA6 was performed using Target Finder and AnnoTALE’s "Predict and Intersect Targets" tool. The EBEs in the *CsGRAS9* promoter are listed in Supplementary Tables S5-8. ( +) indicates the plus strand of the DNA sequence. **B** Prediction of the multichain PthA5–EBE_*proCsGRAS9*_ complex. **C-E** Confirmation of the binding capacity of PthA6 (C), PthA5 (D), and PthA4 (E) to the EBE regions in the *CsGRAS9* promoter using electrophoretic mobility shift assays (EMSA). The probe (*ProCsGRAS9*) is a Cy5-labeled fragment generated from the EBE region of the *CsGRAS9* promoter. Competitive EMSAs showed that purified PthA6-His, PthA5-His, and PthA4-His proteins bound to the Cy5-labeled probes and cold probes (unlabeled competitors). Cold probes acted as competitors at increasing concentrations of 1 × , 100 × , 400 × , and 800 × . The presence or absence of proteins and probes is indicated by " + " or "–". Black arrows denote specific shifts and free probes (below). **F** Yeast one-hybrid (Y1H) assays of PthA5 and PthA6 binding to *CsGRAS9* promoter. The coding sequences of PthA5 and PthA6 were fused to pB42AD, and the *CsGRAS9* promoter was fused to the p*LacZ* vector. Co-transformations of pB42AD-PthA5/pB42AD-PthA6 and p*LacZ* - *proGRAS9* were carried out in yeast strain EGY48. Empty vector p*LacZ* and p*LacZ* - *proGRAS9* were introduced into EGY48 cells carrying pB42AD as negative controls, while the transformants with pB42AD-PthA5/pB42AD-PthA6 and empty vector *pLacZ* were also used as additional negative controls

To investigate the molecular mechanisms of PthA5 and PthA6 in canker formation, we used TALE target prediction tools (AnnoTALE, Target Finder and Talvez) to identify putative EBEs for PthA5 and PthA6 by scanning the sweet orange (*C*. *sinensis*) promoterome (Supplementary Fig. S7 and Tables S5-6). The predicted EBEs for PthA5 and PthA6 were primarily located in the promoter region of *Cs2g22130*, which encodes a putative GRAS family member, *CsGRAS9* (Fig. 2A). Additionally, we analyzed the potential targets of PthA1 and PthA4 in the *CsGRAS9* promoter and observed that the candidate EBE regions displayed features of TATA-like elements (Fig. 2A and Supplementary Tables S7-8), as previously reported (Pereira et al. 2014). To gain further insights into the function of PthA5, we generated a structural model using Alphafold 2, which revealed an uninterrupted right-handed superhelical molecule. PthA5 comprised 19.5 RVD repeats (residues 300 to 908), including a 0.5 repeat at the C-terminus formed by non-conserved amino acids. The 19.5 RVD repeats created a complete helical turn, with the innermost spiral formed by the RVD loops, exhibiting a nearly identical conformation (Fig. 2B). Protein-DNA docking analysis showed a binary complex that the DNA molecule was positioned along the axis within PthA5 superhelical assembly. This structure incorporated the 19.5 RVD repeats and the 27-base pair (bp) DNA-binding element through hydrogen bonds.

We further performed a competitive electrophoretic mobility shift assay (EMSA) to determine whether the PthA4, PthA5, and PthA6 proteins could bind to the predicted EBEs in the *CsGRAS9* promoter. His-tagged fusion proteins PthA6-His, PthA5-His, and PthA4-His were purified, and a 33 bp double-stranded DNA fragment (*ProCsGRAS9*) containing EBEs for PthA4, PthA5, and PthA6 was used as the probe for EMSA (Supplementary Fig. S8 and Fig. 2A). The EMSA results showed that PthA4, PthA5, and PthA6 proteins bound to *ProCsGRAS9*, indicating that the presence of EBEs enabled the specific binding of these TALEs (Fig. 2C-E). Additionally, the binding affinities of these TALEs to the *CsGRAS9* promoter were estimated using the ‘Target Finder’ tool from TAL Effector Nucleotide Targeter 2.0 and the ‘predict and intersect Targets’ feature of AnnoTALE. The analysis demonstrated that PthA1 had the strongest affinity for EBE regions in the *CsGRAS9* promoter, particularly for EBEs G-M and 25–28 (Supplementary Fig. S7B-C) . PthA5 also showed strong affinity for the EBEs, though slightly less than PthA1. In contrast, PthA6 and PthA4 exhibited weaker affinities for the *CsGRAS9* promoter compared to PthA1 and PthA5. Taken together, these findings suggest that among the three effectors, PthA5 exhibits the strongest binding activity to the EBEs in the *CsGRAS9* promoter and plays a pivotal role in regulating *CsGRAS9* expression.

**Fig. 3:**
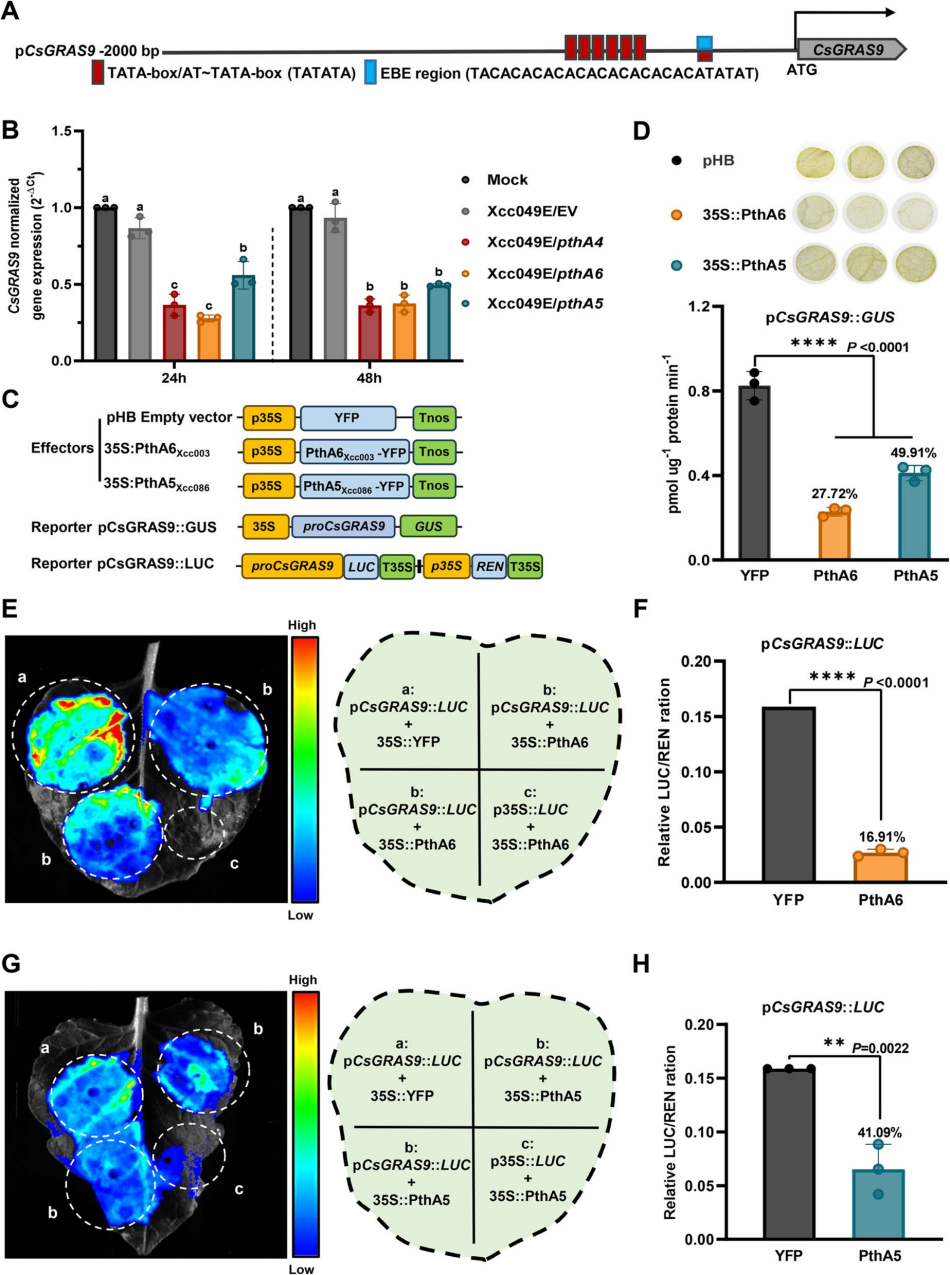
PthA5 and PthA6 regulate the transcription of *CsGRAS9*. **A** An illustration of the 2-kb promoter region of *CsGRAS9* used for yeast one-hybrid (Y1H), glucuronidase (GUS), and luciferase (LUC) assays. Red blocks represent core promoter TATA-box/AT-TATA-box motifs, and blue blocks represent predicted effector binding element (EBE) region. The same components are highlighted with the same color across experiments. **B** Relative expression of *CsGRAS9* in the leaves of grapefruit measured at 24- and 48-h post-inoculation (OD_600_ = 1.0). *CsEf1a* was used as a constitutive reference gene. Three biological replicates are presented as mean ± SEM values. Different letters indicate significant differences (*p* < 0.05) analyzed by two-way ANOVA with Tukey's test. **C** Schematic diagrams of plasmids used in the transient expression assays in *N. benthamiana*. The coding sequences (CDS) of PthA5 and PthA6 were cloned into pHB vector as the effector. A 2000-bp promoter fragment of *CsGRAS9* containing the EBE regions was fused to pCAMBIA1381::GUS and pGreenII0800-LUC vectors to drive the expression of the GUS and LUC reporter genes, respectively. **D** GUS staining and activity assays performed in *N. benthamiana* leaves by infiltration with the labeled effector and reporter combinations. Empty pHB was co-transformed with vector *pCsGRAS9*::*GUS* as the negative control. Results are presented as mean ± SEM values for three biological replicates. Asterisks indicate statistically significant differences using Tukey's test (****p < 0.0001). **E–H** Effector constructs pHB-PthA6, pHB-PthA5 and reporter vector *pCsGRAS9*::LUC were used in transient expression assays to assess luciferase activity. LUC fluorescence images of *N*. *benthamiana* leaves by infiltration with the pHB-PthA6 (**E**), pHB-PthA5 (**G**) and reporter combinations were captured 72 h post-infiltration. Relative luciferase activity of *CsGRAS9* promoter was determined by the ratios of LUC to REN (**F** and** H**). The results show that both PthA6 (**F**) and PthA5 (**H**) repressed *CsGRAS9* transcriptional activity. Mean ± SEM values are shown for three biological replicates. Asterisks denote statistically significant differences with Student’s t-test (****p < 0.0001, **p < 0.01)

In general, *Xanthomonas* TALEs (e.g., AvrBs3, PthXo2, and PthA4) target the EBE regions of promoters containing TATA-box motifs (Wang et al. 2017). We cloned and sequenced the *CsGRAS9* promoter from grapefruit and analyzed it using PlantCARE. Several core TATA-box/AT-TATA-box motifs were identified in the *CsGRAS9* promoter, overlapping with the EBE regions for PthA4, PthA5, and PthA6 (Fig. 3A and Supplementary Fig. S9). A palindromic sequence was also presented in these EBE regions (Supplementary Fig. S7A). To further confirm the interaction of PthA5 and PthA6 with the *CsGRAS9* promoter *in vivo*, we fused a 2k-bp fragment of the *CsGRAS9* promoter to the *pLacZ* reporter vector. As shown in Fig. 2F, PthA5 and PthA6 positively regulated β-galactosidase (*LacZ*) reporter gene expression, demonstrating that PthA5 and PthA6 directly bound to the *CsGRAS9* promoter *in vivo*.

Gene suppression of *CsGRAS9* was observed in Xcc049E/*pthA4*-, Xcc049E/*pthA5*-, and Xcc049E/*pthA6*-infected grapefruit leaves, but not in Xcc049E/EV-infected leaves (Fig. 3B). To confirm that PthA5 inhibits gene expression by binding to the EBEs in the *CsGRAS9* promoter, we used *Agrobacterium*-mediated transient β-glucuronidase (GUS) and luciferase (LUC) expression assays in *N*. *benthamiana*. The full-length *CsGRAS9* promoter was fused with pCAMBIA1381::GUS and pGreenII0800-LUC vector to construct p*CsGRAS9*::*GUS* and p*CsGRAS9*::*LUC*, respectively, where the reporter genes were driven by the *CsGRAS9* promoter (Fig. 3C). As expected, in leaves co-transformed with p*CsGRAS9*::*GUS* and PthA5 or PthA6, we observed a significant reduction in GUS expression, indicating that PthA5 and PthA6 can inhibit *CsGRAS9* expression (Fig. 3D). Histochemical staining also showed that *CsGRAS9* expression was suppressed by PthA5 and PthA6. Similar results were obtained using a transient dual-luciferase assay in *N*. *benthamiana* leaves, where both PthA5 and PthA6 significantly decreased luciferase activity and signal driven by the *CsGRAS9* promoter compared to the pHB empty vector (Fig. 3E-H). These findings indicate that PthA5 and PthA6 directly and negatively regulate *CsGRAS9* expression.

### Natural variation in citrus *GRAS9* promoters and importance of variations in response to *Xcc* infection

**Fig. 4:**
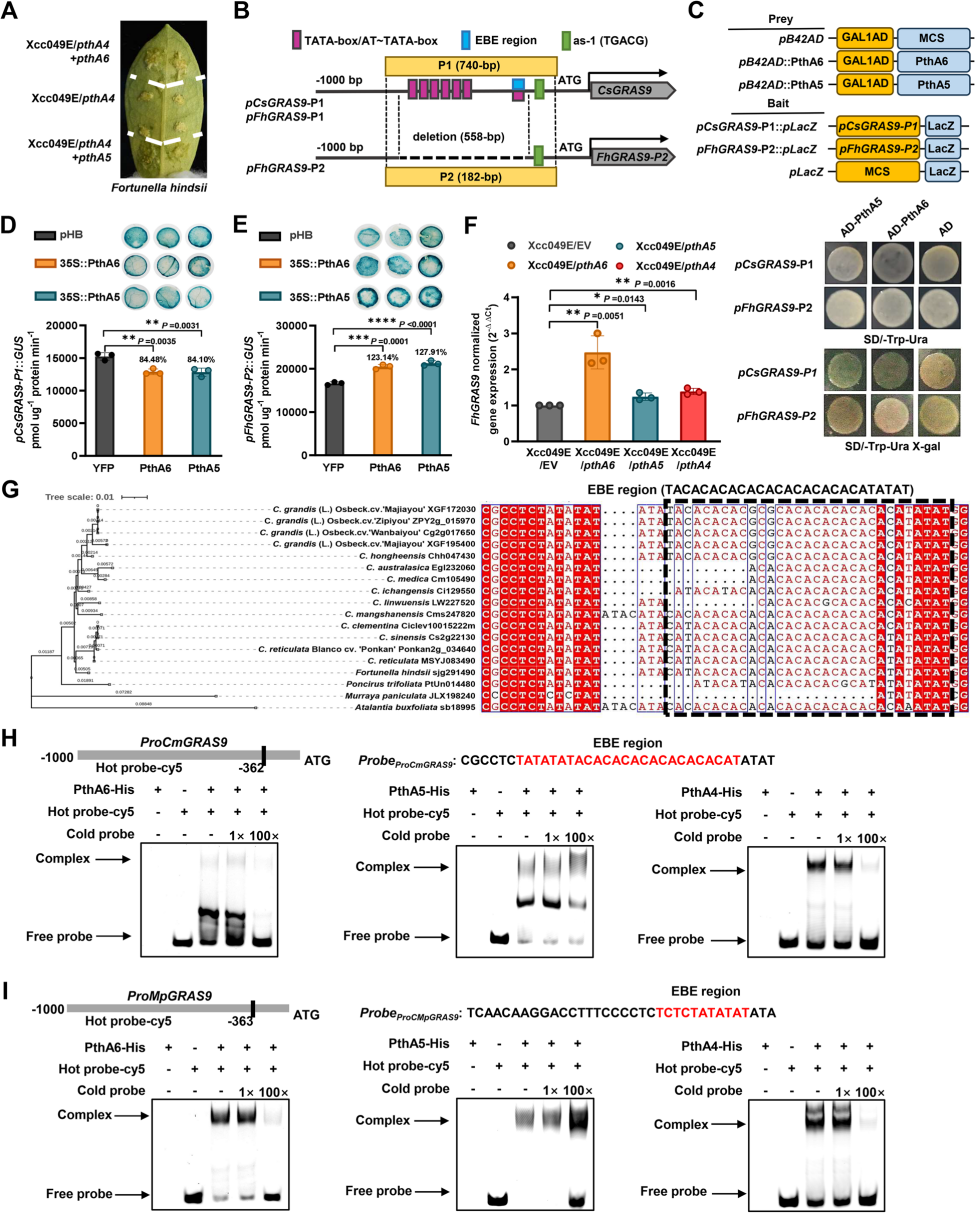
Sequence divergence of *CsGRAS9* in citrus cultivars. **A** Leaves of Hong Kong kumquat (*F*. *hindsii*, Shan Jin Gan) were inoculated with Xcc049E/*pthA4* (control) and a mixed suspension (OD_600_ = 1.0) of Xcc049E/*pthA4* + *pthA5* and Xcc049E/*pthA4* + *pthA6* at a 1:1 ratio. Canker symptoms were observed 16 days post-inoculation. **B** Illustration of the 740-bp promoter region of *CsGRAS9* and the 182-bp promoter region of *FhGRAS9-P2* used for Y1H, GUS, and LUC assays. Pink blocks represent core promoter TATA-box/AT-TATA-box motifs, blue blocks represent predicted EBE regions and green blocks represent as-1 motifs. **C** Schematic of plasmids used in Y1H assays. The CDS of *pthA5* and *pthA6* were cloned into pB42AD vector as the effector, respectively. Truncated promoter fragments of *CsGRAS9* (*CsGRAS9*-P1/*FhGRAS9*-P1) and *FhGRAS9* (*FhGRAS9*-P2) were fused with *pLacZi* reporter genes. Constructs were co-transformed into yeast EGY48 strains, grown on SD/-Leu/-Trp medium, and evaluated on SD/-Leu/-Trp/X-gal plates. Negative controls were co-transformants with empty vectors pB42AD or *pLacZi*. **D-E** GUS staining and expression assays in *N. benthamiana* leaves transformed with labeled effector and reporter constructs via transient expression assays. The CDS of *pthA5* and *pthA6* were cloned into the pHB vector. Truncated promoter fragments (740-bp *CsGRAS9*-P1 in D and 182-bp *FhGRAS9*-P2 in E) were fused with pCAMBIA1381::GUS. Negative controls included empty pHB co-transformed with vectors *pCsGRAS9*-P1::GUS or *pFhGRAS9*-P2::GUS. Three biological replicates were presented as mean ± SEM values. Asterisks indicate statistically significant differences using one-way ANOVA followed by Tukey's test (****p < 0.0001, ***p < 0.001, **p < 0.01). **F** Relative expression of *FhGRAS9* in Hong Kong kumquat (Shan Jin Gan) leaves 48 h post-inoculation (OD_600_ = 1.0). *CsEf1a* was used as a reference gene. Mean ± SEM values from three biological replicates are shown. Asterisks indicate significant differences between bacterial-inoculated leaves using one-way ANOVA with Tukey's test (*p < 0.05, **p < 0.01). **G** Alignment of the allelic variations of the EBE regions of *GRAS9* promoters in citrus species. Sequences show the 26-bp and a 12-bp deletion in the *CsGRAS9* homologs of *M*. *paniculata*, *C*. *australasica* and *C*. *medica*. Conserved nucleotides are in blue, while nucleotide variations are highlighted in black or represented by black dots for missing nucleotides. **H-I** Binding of purified His-tagged proteins PthA6-His, PthA5-His, and PthA4-His to EBE regions in the promoters of *GRAS9* homologs in the *M*. *paniculata*, *C*. *australasica* and *C*. *medica*. EMSA assays were performed with Cy5-labeled probes for *ProCmGRAS9* (**H**) and *ProMpGRAS9* (**I**) and cold competitors. Cold probes were used as competitors with 1 × or 100 × excess over Cy5-labeled probes. Specific shifts and free probes are indicated by arrows. Symbol “ + ” or “–” indicates the presence or absence of protein and probes. Results are from three independent experiments

To verify the role of *GRAS9s* in response to *Xcc* infection, we explored the development of canker symptoms in canker-resistant Hong Kong kumquat (*F*. *hindsii*). When inoculated with both PthA4 and either PthA5 or PthA6, we observed significantly more severe canker symptoms in Hong Kong kumquat compared to those infected with Xcc049E*/pthA4* alone (Fig. 4A). Increased bacterial growth in Hong Kong kumquat was also noted with the presence of PthA5 or PthA6 (Supplementary Fig. S10A). The *GRAS* isoforms from sweet orange and wild tetraploid Hong Kong kumquat were designated *CsGRAS9* and *FhGRAS9*, respectively. To explore the relationship between resistance and promoter polymorphisms, we amplified and sequenced the core 740-bp regions of the *CsGRAS9* and *FhGRAS9* promoters containing EBE regions (Fig. 4B). Sequence alignment revealed a 558-bp deletion (InDel) in the promoter region of *FhGRAS9* (Supplementary Fig. S10B). Different promoter genotypes were identified in Hong Kong kumquat, including a 182-bp fragment (designated *pFhGRAS9*-P2) and a 740-bp fragment identical to the *CsGRAS9* promoter (designated *pCsGRAS9/FhGRAS9-*P1) (Supplementary Fig. S10B-C). The 182-bp *pFhGRAS9*-P2 lacked several AT-TATA/TATA-box and EBE regions for TALEs but contained a cis-element similar to the SA-inducible *activation sequence-*1 (*as*−1). Additionally, the EMSA probe *ProCsGRAS9*, which contains EBEs for PthA4, PthA5, and PthA6, was also detected in the *FhGRAS9-*P1 promoter.

Two truncated plasmids, *pFhGRAS9/CsGRAS9-*P1 (referred to as *pCsGRAS9-*P1) and *pFhGRAS9*-*P2*, were constructed using the *pLacZ* reporter vector. To determine whether the deletion variant in *FhGRAS9* promoter is crucial for interaction with PthA5 and PthA6, we conducted yeast one-hybrid (Y1H) assays in EGY48 strains. The results showed that PthA5 and PthA6 could not target the *FhGRAS9-*P2 promoter to induce *LacZ* reporter gene expression but were able to directly bind to the *FhGRAS9/CsGRAS9*-P1promoter *in vivo* (Fig. 4C). Next, we used *Agrobacterium*-mediated transient GUS expression assays in *N*. *benthamiana* to assess the promoter activities of *pCsGRAS9*-P1 and *pFhGRAS9*-P2. The 740-bp *pCsGRAS9*-P1 and 182-bp *pFhGRAS9*-P2 promoters were fused with the *GUS* reporter vector pCAMBIA1381 to construct *pCsGRAS9*-P1::*GUS* and *pFhGRAS9*-P2::*GUS*, respectively. As shown in Fig. 4D, the relative GUS activity of *pCsGRAS9*-P1 significantly decreased to 84.10% and 84.48% after co-transformation with PthA5 or PthA6, respectively. This result is consistent with findings that PthA5 and PthA6 inhibit *CsGRAS9* expression (Fig. 3D). Conversely, when *pFhGRAS9*-P2::*GUS* was co-infiltrated with pHB-PthA5 or pHB-PthA6 into *N*. *benthamiana*, *GUS* expression increased by 127.91% and 123.14%, respectively, compared to cells co-transformed with the pHB empty vector (Fig. 4E). Histochemical staining also confirmed that PthA5 and PthA6 suppressed the promoter activity of *FhGRAS9/CsGRAS9*-P1 but not that of *FhGRAS9*-P2 (Fig. 4D and E). Consistent with the quantitative analysis of *GUS* activity, expression analysis indicated that PthA4, PthA5, and PthA6 induced the *FhGRAS9* expression in Hong Kong kumquat leaves (Fig. 4F). These findings suggest that natural variation in the *FhGRAS9* promoter disrupts its interaction with TALEs, leading to increased *FhGRAS9* expression.

Citrus germplasm, which includes wild species resistant to citrus canker, provides an abundant genetic resource for unraveling the molecular mechanisms of plant-microbe interactions (Duan et al. 2022). The genome of 14 citrus species, including *C*. *grandis*, *C*. *hongheensis*, *C*. *australasica*, *C*. *medica*, *C*. *ichangensis*, *C*. *linwuensis*, *C*. *mangshanensis*, *C*. *clementina*, *C*. *sinensis*, *C*. *reticulata*, *F*. *hindsii*, *Murraya paniculata*, *Poncirus trifoliata*, and *A. buxifolia*, are available in the Citrus genome database (http://citrus.hzau.edu.cn/orange/). To investigate whether natural variations in the *GRAS9s* promoters contribute to differential canker symptoms in citrus plants, we retrieved the 2000 bp *GRAS9* promoter sequences from the Citrus genome database. Alignment of these promoters revealed that InDel and SNP variations were concentrated in the EBE regions, causing mismatches between RVDs and EBEs (Fig. 4G). Specifically, 26-bp, 12-bp, and 10-bp deletions were detected in the *GRAS9* promoters of* C*. *medica*, *M*. *paniculata*, *C*. *australasica*, and *C*. *linwuensis*, respectively. Phylogenetic analysis of the *GRAS9* promoters showed that the TALE-targeted EBE regions in* C*. *sinensis* clustered with those in susceptible species like *C*. *clementina* and *C*. *reticulata* (Fig. 4G). *C*. *medica* has been identified as strongly resistant to *Xcc* (Fu et al. 2020), while orange jessamine (*M*. *paniculate*) is a non-host of *Xcc*, displaying only faint chlorosis as the most severe symptom at 26 dpi (Ference et al. 2020). To validate the binding efficiency of PthA4, PthA5, and PthA6 with the *C*. *medica* and *M*. *paniculate GRAS9* promoters, we performed EMSA assays using two 35 bp double-stranded DNA fragments (*ProCmGRAS9* and *ProMpGRAS9*) containing sequences aligned with EBEs for TALEs (Fig. 4H and I). EMSA results confirmed that natural deletion variations in the EBE regions of the *CmGRAS9* and *MpGRAS9* promoters slightly affected the binding efficiency of PthA4, PthA5, and PthA6 (Fig. 4H and I). These findings suggest that resistance to citrus canker is associated with natural variations in the EBE regions of the *GRAS9s* promoters, which influence the expression patterns of *GRAS9* under *Xcc* infection.

### TALEs suppress disease resistance to citrus canker through regulating *CsGRAS9* expression

**Fig. 5:**
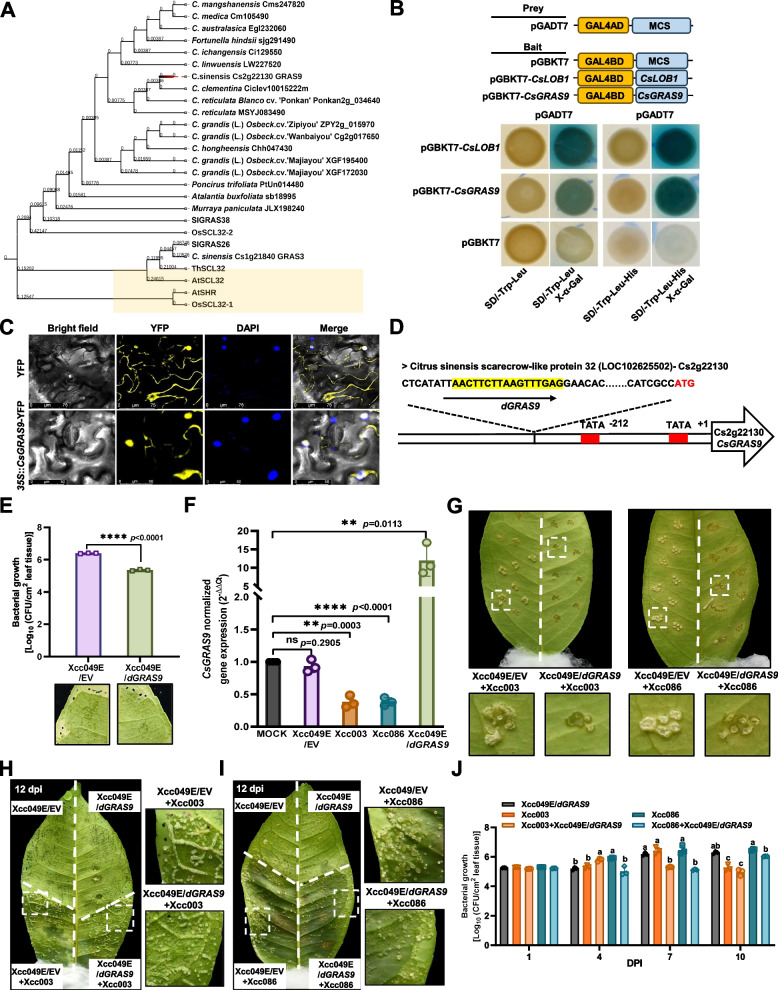
Subcellular localization, transcriptional activities and function of *CsGRAS9*. **A** Phylogenetic tree of CsGRAS9, its closest homolog (CsGRAS3), homologous proteins in *Citrus spp*., and orthologous proteins in *Arabidopsis*, rice, and tomato, constructed using MEGA X. Branch lengths represent the number of substitutions per site. **B** Analysis of *CsGRAS9* transcriptional activity in yeast. The CDS of *CsLOB1* and *CsGRAS9* were fused to pGBKT7 vector, respectively. The pGBKT7-*CsGRAS9* construct was co-transformed with pGADT7 into yeast strain AH109. Yeast cells expressing BD-CsGRAS9 were spotted on SD/-Trp/-Leu, SD/-Trp/-Leu/-His media, and selection media containing X-*α*-gal. pGBKT7-*CsLOB1* and pGADT7 served as positive and negative controls, respectively. **C** Subcellular localization of CsGRAS9 in *N. benthamiana* leaves. CsGRAS9 was fused with YFP in the pHB vector (35S::*CsGRAS9*-YFP). Fluorescence (yellow for YFP, blue for DAPI) was observed using a confocal microscope. Scale Bars: 50 and 75 μm.** D** *CsGRAS9* specifically activated by dTALEs. The region targeted by dGRAS9 in the *CsGRAS9* promoter is highlighted in yellow. Red nucleotides represent the translation start site. **E** Grapefruit leaves were infiltrated with *Xcc* suspensions (OD_600_ = 0.6) of Xcc049E/EV and Xcc049E/*dGRAS9*. Citrus canker symptoms and bacterial growth were evaluated at 16 days post-inoculation. Error bars represent the standard deviation of three biological replicates. Asterisks indicate statistically significant differences (****p < 0.0001) using Student’s t-test. **F** Relative expression of *CsGRAS9* in grapefruit leaves was measured 48 h post-inoculation (OD_600_ = 1.0). *CsEf1a* was used as the internal control. Data from three biological replicates are presented as mean ± SEM values. Asterisks denote significant differences using Student’s t-test (*p < 0.05, **p < 0.01, ****p < 0.0001). **G** Grapefruit leaves were inoculated using a pinprick method with *Xcc* suspensions (OD_600_ = 1.0) of mixed suspensions Xcc003 + Xcc049E/EV, Xcc003 + Xcc049E/*dGRAS9*, Xcc086 + Xcc049E/EV, and Xcc086 + Xcc049E/*dGRAS9* in a 1:1 ratio. Positive controls included inoculations with Xcc003 + Xcc049E/EV and Xcc086 + Xcc049E/EV suspensions. Citrus canker symptoms were assessed 8 days after inoculation. **H-I** Grapefruit leaves were infiltrated with *Xcc* suspensions (OD_600_ = 1.0). Xcc049E/EV and Xcc049E/*dGRAS9* inoculations were performed, alongside Xcc003 + Xcc049E/EV (**H**) and Xcc086 + Xcc049E/EV (**I**) inoculations, to evaluate citrus canker severity. Symptoms were assessed 12 days post-inoculation. **J** Bacterial growth of *Xcc* strains in grapefruit leaves was monitored at 1, 4, 7, 10 days post-inoculation. Data are presented in mean colony-forming units (CFU)/cm^2^. Error bars represent standard deviations from three biological replicates. Different letters denote statistically significant differences using two-way ANOVA with Tukey's test (*p* < 0.05)

Considering that TALEs inhibited *CsGRAS9* homologs expression in susceptible citrus plants, but not in kumquat, we further examined whether *GRAS9* expression attributed to citrus canker resistance. First, we obtained the coding sequence (CDS) of *CsGRAS9* from grapefruit leaves. Sequence analysis revealed that the *CsGRAS9* transcript was 1345 bp, encoding a 49.7 kDa protein with 448 amino acids. Among the GRAS family proteins in citrus, CsGRAS9 is most closely related to CsGRAS3 (Zhang et al. 2019). Phylogenetic analysis of CsGRAS9 and its orthologs in various citrus species, as well as tomato, rice, *Arabidopsis*, and *Tamarix hispida*, was conducted by aligning amino acid sequences. CsGRAS9 clustered in the same clade as known plant development-related GRAS proteins, including *Arabidopsis* AtSCL32, tomato SlGRAS38 and SIGRAS26, *Tamarix hispida* ThSCL32, and rice OsSCL32-2 (Fig. 5A). Multiple sequence alignment further revealed high amino acid identity among citrus GRAS9 proteins (Supplementary Fig. S11), indicating evolutionary conservation across citrus species. CsGRAS9 shared the highest homology with GRAS9 from *C*. *clementina* and *C*. *reticulata*. Moreover, the MEME suite (http://meme-suite.org/tools/meme) identified 20 conserved motifs in GRAS9 proteins of citrus species and their orthologs in other plants. In general, GRAS9 proteins within the same clade contain similar motifs. Motifs 1–14 and motifs 16–17 were conserved in citrus GRAS9 proteins, while three motifs (motifs 1, 3, and 11) and four motifs (motifs 2, 9, 11, and 13) were also found at the N-terminal and C-terminal regions of SlGRAS26, SlGRAS38, CsGRAS3, ThSCL32, and OsSCL32-2 (Supplementary Fig. S12). To assess whether CsGRAS9 has transcriptional activity, *CsGRAS9* and *CsLOB1* were cloned into the pGBKT7 vector. The BD-CsGRAS9 and BD-CsLOB1 constructs, along with the empty vector BD, were individually co-transformed with pGADT7 into yeast strain AH109. Yeast cells carrying BD-CsLOB1 and BD served as positive and negative controls, respectively. Yeast cells carrying BD-CsGRAS9 grew and turned blue on SD/−Trp/−Leu/X-α-Gal and SD/-Trp-Leu-His/X-α-Gal selection medium, indicating that CsGRAS9 possesses transcriptional activity (Fig. 5B). Subcellular localization of CsGRAS9 was determined by transfecting a CsGRAS9-GFP fusion construct into *N. benthamiana* leaves. Fluorescence signals in *N. benthamiana* overexpressing CsGRAS9-GFP were detected in the nucleus and cytosol (Fig. 5C).

A designer transcription activator-like effector (dTALE), designated dGRAS9, was constructed to target the 17-bp EBE of the *CsGRAS9* promoter located 212 bp upstream of the ATG start codon (Fig. 5D). The EBE targeted by dGRAS9 is positioned 1 bp upstream of the predicted ATTATA and TATA-box in the *CsGRAS9* promoter. Expression of the dGRAS9 protein in Xcc049E strains harboring *dGRAS9* (Xcc049E/*dGRAS9*) was confirmed by western blotting using an anti-flag antibody (DYKDDDDK), with the dGRAS9 protein appearing at the expected size of 120.2 kDa (Supplementary Fig. S13). In grapefruit leaves, bacterial growth was reduced in Xcc049E/*dGRAS9* -infected plants compared to Xcc049E/EV, within 18 dpi, suggesting that *CsGRAS9* plays a role in plant resistance (Fig. 5E). Additionally, Xcc049E/*dGRAS9* activated *CsGRAS9* expression within 48 h post-inoculation (Fig. 5F).

To further investigate whether *CsGRAS9* contributes to citrus canker resistance, we observed canker symptom development in grapefruit leaves inoculated via the pinprick and infiltration methods using mixed bacterial suspensions of Xcc003/EV or Xcc086/EV combined with Xcc049E/*dGRAS9* at a 1:1 ratio. Inoculation with Xcc003/EV + Xcc049E/*dGRAS9* and Xcc086/EV + Xcc049E/*dGRAS9* suppressed canker symptom development compared with the controls (Fig. 5G-I and Supplementary Fig. S14). Consistent with these findings, reduced bacterial growth in grapefruit leaves, induced by *CsGRAS9* expression, confirmed that *CsGRAS9* plays a role in citrus canker resistance (Fig. 5J).

### Evaluation of CsGRAS9 mutants

**Fig. 6:**
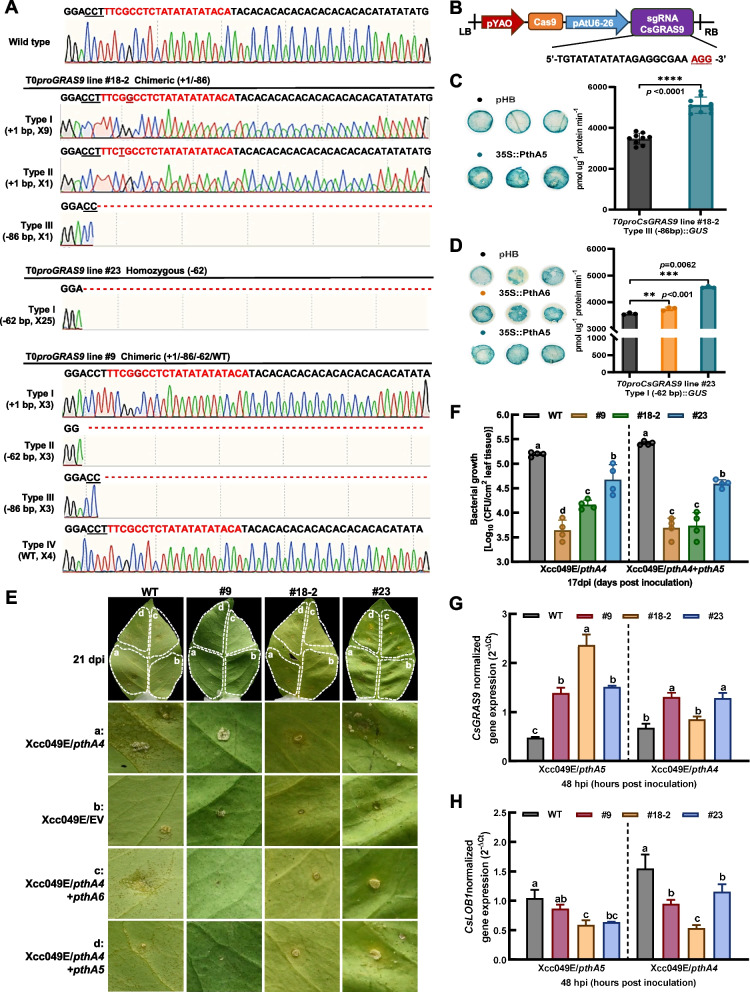
*CsGRAS9* mutants of *Citrus sinensis* cv. 'Anliu' sweet orange generated by genome editing of the promoter of *CsGRAS9*. **A** Chromatograms of PCR products from wild-type (WT) *C. sinensis* cv. 'Anliu' sweet orange serve as the negative control. Colony sequencing chromatograms from the T0 *proCsGRAS9*-edited *C. sinensis* cv. 'Anliu' line #18–2 show three types of insertions and deletions at the target site: Type I (+ 1 bp insertion), Type II (+ 1 bp different insertion), and Type III (−86 bp deletion). Representative chromatograms are shown. In line #23, mutations in both alleles of the *CsGRAS9* promoter are shown, with a 62 bp deletion as the only type of mutation. Colony sequencing chromatograms from line #9 show four types of mutations: Type I (+ 1 bp insertion), Type II (−62 bp deletion), Type III (−86 bp deletion), and Type IV (WT). EBE regions are highlighted in red. “–” indicates deletions, “ + ” indicates insertions. The underlined black (CCT) indicates PAM. **B** Schematic representation of the CRISPR construct containing the *CsGRAS9* gRNA. The purple block indicates the sgRNA scaffold, and red nucleotides were chosen for the gRNA in the EBE region of the *CsGRAS9* promoter; the protospacer-adjacent motif (PAM) is underlined. **C-D** GUS staining and expression in *N. benthamiana* leaves transformed with effector and reporter combinations via transient expression assays. The CDS regions of *pthA5* and *pthA6 *were cloned into the pHB vector as effectors. Truncated promoter fragments of the 604-bp Type III (−86 bp) promoter from T0 *proCsGRAS9* line #18–2 were fused to pCAMBIA1381::GUS. Effector construct pHB-*pthA5* and reporter vector T0 *proCsGRAS9* line #18–2 Type III (−86 bp)::GUS were used in GUS assays (**C**). Asterisks indicate statistically significant differences (****p < 0.0001) using Student’s t-test. Truncated promoter fragments of the 628-bp Type I (−62 bp) promoter from T0 *proCsGRAS9* line #23 were fused to pCAMBIA1381::GUS. Effector constructs pHB-*pthA5* or pHB-*pthA6* and reporter vector T0 *proCsGRAS9* line #23 Type I (−62 bp)::GUS were used for GUS assays (**D**). Asterisks indicate statistically significant differences by one-way ANOVA with Tukey’s test (**p < 0.01, ***p < 0.001). **E** Leaves of WT and T0 *proCsGRAS9*-edited *C. sinensis* cv. 'Anliu' lines (#9, #18–2, and #23) were infiltrated with *Xcc* suspensions (OD_600_ = 1.0) of Xcc049E/EV, Xcc049E/*pthA4*, and mixed suspensions of Xcc049E/*pthA4* + *pthA5* and Xcc049E/*pthA4* + *pthA6* in a 1:1 ratio. Citrus canker symptoms were evaluated 21 days after inoculation. **F** Bacterial growth of *Xcc* strains in the leaves of WT and *proCsGRAS9* mutants of 'Anliu' sweet orange was monitored 17 days post-inoculation. Error bars represent standard deviations of three biological replicates. Different letters indicate statistically significant differences by two-way ANOVA with Tukey’s test (*p* < 0.05). **G-H** Relative expression of *CsGRAS9* (**G**) and *CsLOB1* (**H**) in leaves of WT and T0 *proCsGRAS9*-edited *C. sinensis* cv. 'Anliu' lines (#9, #18–2, and #23) measured 48 h post-inoculation (OD_600_ = 1.0). *CsEf1a* was used as a constitutive standard. Different letters indicate statistically significant differences by two-way ANOVA with Tukey’s test (*p* < 0.05)

Natural variations in the promoters of *GRAS9* genes are associated with their expression and resistance to *Xcc* infection in citrus germplasms. To explore the role of *CsGRAS9* in canker resistance, several mutations in the *CsGRAS9* promoter (*proCsGRAS9*) were generated using the PTG/Cas9 system (Fig. 6A). One sgRNA was specifically designed to target the EBE regions of the *CsGRAS9* promoter (Fig. 6B). The recombinant vector pCAMBIA1300-pYAO-Cas9/pGRAS9 was introduced into ‘Anliu’ sweet orange epicotyl via *Agrobacterium*-mediated transformation. As a result, 19 eGFP-positive T0 lines were successfully regenerated and survived in the greenhouse. Among these, three lines were identified as wild-type (Fig. 6A). Hi-Tom sequencing of the 19 regenerated lines tentatively revealed that 9 of them were homozygous mutants. Sweet orange has two alleles, each with conserved EBEs in the promoter regions. Sanger monoclonal sequencing confirmed the mutant genotypes in both alleles of the edited lines. Sequence chromatograms at the target sites showed that the most frequent mutation was a 1 bp insertion. Line #23 was identified as a homozygous mutant (−62/−62), featuring a 62 bp deletion in the EBE regions of the *CsGRAS9* promoter (Fig. 6A). Among the two chimeric *proCsGRAS9*-edited mutants, line #18-2 exhibited three mutation types (−86/+1/+1), while line #9 had four mutation types (+1/−86/−62/WT) at the target site (Fig. 6A). Additionally, line #13, a biallelic *proGRAS9* mutant (−7/−9), had 7 bp and 9 bp deletions upstream of the EBE regions for TALEs (Supplementary Fig. S15A). To assess potential off-target effects, two possible off-target sites containing up to four mismatches were identified in the targeted *CsGRAS9* promoter using CRISPR P v2.0. Sanger sequencing of the off-target regions in edited lines (#9, #13, #18-2, and #23) revealed no off-target mutations (Supplementary Fig. S16).

To further validate the role of natural variations in promoter activity, *CsGRAS9* promoters from three *proGRAS9* lines (#18-2, #23, and #9) were cloned into the pCAMBIA1381::GUS vector. Constructs *pCsGRAS9* Type I (−62 bp)::*GUS*, *pCsGRAS9* Type III (−86 bp)::*GUS*, and *pCsGRAS9* Type I (+1 bp)::*GUS* were co-infiltrated with PthA5 or PthA6 into *N. benthamiana* leaves. The results showed higher *GUS* activity for the *pCsGRAS9* Type I (−62 bp) and Type III (−86 bp) promoters, while decreased *GUS* activity was observed for the *pCsGRAS9* Type I (+1 bp) promoter, which was consistent with wild-type levels (Fig. 6C and D, and Supplementary Fig. S17). These findings indicated that gene-edited variations in the *CsGRAS9* promoter can regulate *CsGRAS9* transcription by disrupting the TALE-EBE interactions. Overexpression of *CsGRAS9* suppressed the development of canker symptoms (Fig. 5). As expected, typical canker symptoms were observed in wild-type ‘Anliu’, while only slight lesions appeared in the *proGRAS9* lines (#9, #18-2, and #23) following *Xcc* infection via infiltration (Fig. 6E and Supplementary Fig. S18). Correspondingly, bacterial titers of *Xcc* were significantly reduced in the *proGRAS9* lines (#9, #18-2, and #23) compared to those in wild-type ‘Anliu’ (Fig. 6F). Interestingly, canker symptoms in the *proGRAS9* line #13 were similar to those in the wild-type (Supplementary Fig. S15B), which is consistent with previous studies that typically excluded chimeric-edited lines due to suboptimal editing types (Yu et al. 2024). Despite this, stable *CsGRAS9* expression and canker resistance were observed in the chimeric-edited line #9 (including the wild-type segment) (Supplementary Fig. S19). PthA4 and PthA5 downregulated *CsGRAS9* expression during *Xcc* infection (Fig. 3B). However, mutations in the EBE regions of the *CsGRAS9* promoter prevented the suppression of *CsGRAS9* by Xcc049E/*pthA4* and Xcc049E/*pthA5* (Fig. 6G). Additionally, the expression of *CsLOB1* was significantly lower in the *proGRAS9* mutants (#9, #18-2, and #23) than in the wild type (Fig. 6H). These alterations in *CsGRAS9* and *CsLOB1* expression help explain the reduced canker symptoms observed in *proGRAS9-*edited lines.

### CsGRAS9 regulates the expression of *CsTAC1* and GA-related genes

To explore the biochemical functions of *CsGRAS9* and map its molecular interaction network, we used STRING to identify protein-protein interactions (PPIs) (Supplementary Fig. S20A). We identified nine proteins interacting with CsGRAS9, including NAC domain-containing protein 33 (SMB/NAC33, orange1.1t03221), Dof zinc finger protein DOF4.6 (DOF4.6, Cs5g01740), Calmodulin-like protein 30 (CML30, Cs5g28300), TELOMERASE ACTIVATOR1 (TAC1, Cs5g06630), LOB DOMAIN-CONTAINING PROTEIN 29 (ASL16/LBD29, Cs5g30050), LOB DOMAIN-CONTAINING PROTEIN 16 (LBD16, Cs5g30060), BOLA-LIKE PROTEIN 1 (BolA1, orange1.1t02810) and two DELLA proteins, Scarecrow-like protein 22 (SCL26/GRAS23, Cs6g09040), and MYCORRHIZ A-INDUCED GRAS (MIG1, orange1.1t01684). Quantitative real-time PCR (qRT-PCR) assays showed that *CsGRAS9* activated the expression of *DOF4.6* and *CML30*, while repressing the expression of *TAC1* (Supplementary Fig. S20B and C). To validate the interaction between CsGRAS9 and CsTAC1, we performed a yeast two-hybrid (Y2H) analysis. Yeast strains co-transformed with AD-CsGRAS9 and BK-CsTAC1 grew on selective medium (SD/-Trp/-Leu/-His/-Ade) and exhibited *α*-galactosidase activity, confirming that CsGRAS9 interacted with CsTAC1 that is the orthologous to AtTAC1, which responses to auxin signaling and induce telomerase activity in *Arabidopsis thaliana* (Supplementary Fig. S20D). Studies have shown that DELLA proteins act as repressors of the gibberellin (GA) signaling pathway (Neves et al. 2023). Infection by *X. campestris* pv. *vesicatoria* (*Xcv*) triggers defense responses by inducing the expression of GRAS subfamily members, such as *SlGRAS4*, *SlGRAS13*, and *SlGRAS6*. To investigate the role of *CsGRAS9* in the regulation of GA biosynthesis and metabolism during Xcc049E/*dGRAS9* infection, we assessed the expression of key GA-related genes. The expression of the GA biosynthetic enzyme GA3 oxidase genes (*GA3ox2* and *GA3ox3*, encoded by* Cs4g20350* and *Cs4g17110*, respectively) and GA20 oxidase 1 (*GA20ox1*, encoded by *Cs1g09880*) were upregulated in *CsGRAS9*-overexpressing leaves (Supplementary Fig. S21A-C). In contrast, the expression of GA methyltransferase *GAMT2* (*Cs9g03520*) was reduced by approximately 2-fold (Supplementary Fig. S21D). These findings suggest that *CsGRAS9* may contribute to citrus canker resistance by modulating the expression of *CsTAC1* and GA-related genes, potentially influencing the GA signaling pathway.

## Discussion

*Xanthomonas* TALEs undergo evolutionary changes in response to selective pressures exerted by their hosts (Gochez et al. 2018). The evolutionary dynamics of the LJ207-7 strain indicated that genetically monomorphic bacteria adapt to environmental changes and diverse hosts through the horizontal gene transfer of TALEs (Ruh et al. 2017; Richard et al. 2017). To further investigate this adaptive mechanism, in the present study, we sequenced the complete *pthA5* and *pthA6* genes to determine their target specificities and shed light on pathogen population behavior and host aggressiveness (Fig. 1 and Supplementary Figs. 1–3). These findings support the hypothesis that recombination and horizontal gene transfer drive convergent evolution among TALEs in *Xanthomonas* strains (Gochez et al. 2018). Understanding the target specificity of TALEs and mechanisms underlying the regulation of host susceptibility or resistance is critical for breeding citrus plants with stable resistance to pathogens.

FuncTAL and DisTAL categorize *Xanthomonas* diversity on the basis of functional binding specificities and ecological diversity, as determined by RVD alignment (Pérez-Quintero et al. 2015). In the present study, FuncTAL analysis revealed that PthA5 and PthA6 share functional similarities with PthA1 (Supplementary Fig. S2). After infection with Xcc049E/*pthA5*, the expression of *CsLOB2* and *CsDIOX* increased slightly (Supplementary Fig. 5), consistent with their upregulation in response to PthA1 (Abe et al. 2016). Moreover, PthA5 and PthA6 promoted the formation of water-soaked lesions and bacterial colonization in grapefruit leaves, independent of *CsLOB1* activation (Supplementary Fig. 4). Typical hypertrophy and hyperplasia were observed after inoculation with Xcc049E/*pthA5* and Xcc049E/*pthA6*, highlighting the need to further investigate mechanisms underlying the actions of PthA5 and PthA6. In grapefruit, a susceptible genotype, the expression level of *CsGRAS9* was suppressed following *Xcc* inoculation, whereas that of *FhGRAS9*, the homolog in Hong Kong kumquat, was expressed (Fig. 4F). This differential response suggests a key role of *GRAS9* homologs in citrus resistance mechanisms.

*Xanthomonas* species enhance bacterial virulence and promote symptom formation by regulating the expression of TALE-targeted genes (Timilsina et al. 2020). Several TALEs, including PthA4, PthAW, PthA∗, PthB, and PthC consistently recognize *CsLOB1* during plant–pathogen interactions, following a well-characterized mechanism (Teper et al. 2020). Deletion events within the repeat arrays of TALEs do not disrupt their conserved functions (Ji et al. 2016; Read et al. 2016). Our findings indicate that both PthA5 and PthA1 may bind to more than four EBEs in the *CsGRAS9* promoter, overlapping with EBEs for PthA4 and PthA6 (Fig. 2A). Protein-DNA docking, which is widely used to predict DNA-binding site architecture, revealed the interactions of TALEs with their target DNA (Deng et al. 2012). The crystal structures of *Xanthomonas* TALEs bound to DNA have established a foundation for exploring TALE functions with high modularity (Li et al. 2023). Structural analysis confirmed that the recognition region of PthA5, composed of 17 direct repeats, specifically binds to the sense strand of the *CsGRAS9* promoter’s double-stranded DNA along the major groove (Fig. 2B). For example, residues N300 and I301 in PthA5 repeat 1 recognize adenine (A) through van der Waals interactions. These physical interactions between PthA5 or PthA6 and the EBE regions of the *CsGRAS9* promoter effectively repress *CsGRAS9* transcription, thereby promoting bacterial pathogenicity. Palindromic sequences in the EBE region might interfere with transcription initiation (Ueda et al. 2020; Luo et al. 2022). However, additional data are required to confirm this mechanism. Moreover, *CsNCED1* (*Cs5g14370*) and *CsABA2* (*Cs6g19380*) were identified as potential target genes of PthA5 (Supplementary Table S5), indicating the complexity of the relationship between TALEs and host gene regulation. These results demonstrate that the interaction between TALEs and host genes is more complex and multifaceted than previously anticipated.

GRAS family members play crucial roles in plant growth and development as well as in various physiological processes, including GA and phytochrome signaling, responses to abiotic and biotic stresses, and chlorophyll biosynthesis (Lu et al. 2022, Neves et al. 2023). For example, *GhSCL13*-*2A* enhanced resistance to *Verticillium dahliae* in cotton by regulating jasmonic acid (JA) and salicylic acid (SA) signaling pathways and reactive oxygen species (ROS) accumulation (Chen et al. 2023). Similarly, *MeDELLAs* improve the resistance of cassava to bacterial blight caused by *Xanthomonas axonopodis* pv. *manihotis* (*Xam*) infection (Li et al. 2018). Moreover, grapevine (*Vitis vinifera* L.) *VviSHR5* expression was induced by *Botrytis cinerea* infection (Grimplet et al. 2016). In rice, three homologs of *CsGRAS9* (*OsGRAS8*, *OsGRAS39*, and *OsSHR1*) confer resistance to bacterial leaf blight (BLB) caused by *Xanthomonas oryzae* pv. *oryzae* (*Xoo*) and sheath blight caused by *Rhizoctonia solani* (Dutta et al. 2021). In the present study, phylogenetic analysis revealed that CsGRAS9 was closely related to SCL32 subfamily members (AtSCL32, SlGRAS38, SlGRAS26, ThSCL32, and OsSCL32-2). In *Tamarix hispida*, *ThSCL32* regulates ROS accumulation by enhancing antioxidant enzyme activity (Lei et al. 2023). To investigate the role of *CsGRAS9* in canker resistance, we induced gene expression of *CsGRAS9* by Xcc049E/*dGRAS9* (Fig. 5). Overexpression of *CsGRAS9* led to delayed pustule formation, decreased development of canker symptoms, and repression of *CsLOB1* expression. These findings indicate that *CsGRAS9* promotes resistance to citrus canker.

In response to *Pseudomonas syringae* pv. *actinidiae* infection, resistant plant varieties exhibit increased expression of resistance-related genes, whereas susceptible varieties display decreased expression (Zhao et al. 2024). The protein structures of citrus *GRAS9* homologs were found to be remarkably conserved (Supplementary Fig. 11). *F*. *hindsii* demonstrates durable resistance to canker disease through PCD and ROS accumulation while also inducing defense-related gene expression (Wu et al. 2018; Zhu et al. 2019). In the present study, we identified transcriptional differences between *CsGRAS9* and *FhGRAS9* during *Xcc* infection in citrus plants (Fig. 4). In addition, we detected a natural variation involving a 558-bp deletion in the *FhGRAS9* promoter (Supplementary Fig. 10B). PthA5 and PthA6 significantly inhibited the transcriptional activity of the *CsGRAS9*-P1 promoter but did not affect the *FhGRAS9*-P2 promoter. This finding is consistent with the observed differences in *CsGRAS9* and *FhGRAS9* expression levels following *Xcc* infection (Fig. 4D–F). However, the presence of a genotype matching the *CsGRAS9* promoter suggests that multiple TALEs, including PthA4, PthA5, and PthA6, collaboratively regulate *FhGRAS9* expression, resulting in enhanced citrus canker symptoms. Similarly, natural variation in the EBE regions of the *AbLOB1* promoter in *A. buxifolia* (primitive citrus) affects *AbLOB1* expression (Tang et al. 2021b). On the basis of these findings, we speculate that mutations in promoter regions stabilize *FhGRAS9* expression and increase canker resistance in *F. hindsii*. In addition, Citron C-05 (*C*. *medica*) exhibits resistance to citrus canker by restricting *Xcc* proliferation (Fu et al. 2020), and orange jessamine (*M*. *paniculata*) shows faint chlorosis with low *Xcc* populations (Ference et al. 2020). Natural genetic variation improves growth-defense trade-offs, reflecting the adaptation of plants to diverse stress pressures (He et al. 2022). In the present study, we identified multiple sequence variations in the *GRAS9* promoters of orange jessamine and citron but not in susceptible citrus biotypes. These insertions and deletions caused frameshifts that slightly affected the binding of TALE to the EBEs of *CmGRAS9* and *MpGRAS9* (Fig. 4H–I). Future experiments are required to validate the hypothesis that natural mutations of *GRAS9s* promoter in *C*. *medica* and *M*. *paniculate* could evade the inhibitory effects of TALEs on *GRAS9s* gene expression by obstructing TALEs from binding to the EBE regions in plants. These findings suggest that natural variations in the *GRAS9* promoters among citrus germplasms confer resistance to different *Xcc* races.

CRISPR/Cas9 systems have been widely used to elucidate gene function by enabling efficient genetic modifications in fruit tree species, such as ‘Anliu’ sweet orange and grape (Tang et al. 2021b, Ren et al. 2021). Additionally, genome editing has been applied to modify the EBE regions in gene promoters, creating plants resistant to bacterial and fungal pathogens, including citrus canker (Su et al. 2023), BLB (Xu et al. 2019), and powdery mildew (Li et al. 2022a, b). In this study, we employed the PTG/Cas9 system to modify the EBE regions in the *CsGRAS9* promoter (Fig. 6B). Multiple homozygous and biallelic (chimeric) sweet orange lines with targeted mutations in the EBE regions were generated (Fig. 6A). Consistent with previous studies, the predominant mutation types in citrus were 1-bp A/T base insertions or deletions (Xu et al. 2022). No off-target mutations were observed in the *CsGRAS9*-edited lines (Supplementary Fig. 16). We also assessed *CsGRAS9* expression in wild-type and *proGRAS9*-edited lines (#9, #18-2, and #23) after inoculation with the control and the *tal*-free strain Xcc049E/EV. As shown in Supplementary Fig. S19, a slight change in *CsGRAS9* expression was observed in chimeric-edited line #18-2, which exhibited an 86-bp deletion. Furthermore, both alleles of the homozygous line #23 contained the same 86-bp deletion, significantly reducing *CsGRAS9* expression. In line with this, *CsGRAS9* expression in these lines was altered by Xcc049E/EV infection, correlating with increased resistance to citrus canker. Editing the promoter region effectively abolished the inhibition of *CsGRAS9* by Xcc049E/*pthA4* and Xcc049E/*pthA5* (Fig. 6G). Modifying the EBEs in *CsGRAS9* promoter delayed canker symptom development and decreased *Xcc* growth in host, consistent with the observed symptoms under Xcc049E/*dGRAS9* infection (Fig. 6F). These results suggest that CRISPR/Cas9 system is an efficient tool for studying the *Xcc*/citrus pathosystem and offers a sustainable approach to managing citrus canker. Furthermore, our findings support the hypothesis that mutations in the EBE regions of *CsGRAS9* promoter enhance resistance to citrus canker.

Previous studies have shown that GRAS proteins play a key role in disease resistance by regulating GA biosynthesis and signaling pathways (Zhou et al. 2018). For example, XopD_Xcc8004_ interfered with DELLA degradation, thereby suppressing GA signaling and promoting disease tolerance (Tan et al. 2014). In rice, overexpression of *OsGA20ox3* leads to increased GA accumulation and heightens the susceptibility of wild-type plants to *Xoo* and *M*. *oryzae* (Qin et al. 2013). Our research demonstrated that overexpression of *CsGRAS9* in grapefruit elevated the transcript levels of GA biosynthetic genes (*GA20ox1*, *GA3ox2* and *GA3ox3*) while reducing the expression of GA methyltransferase 2 (*GAMT2*) (Supplementary Fig. S21A-D). These findings suggest that *CsGRAS9* promotes the expression of GA biosynthetic genes, potentially revealing an adaptive mechanism that fine-tunes GA signaling to balance defense responses in citrus. In summary, our findings allowed us to put forward a model of how TALEs promote bacterial virulence and how *CsGRAS9* regulates citrus canker resistance (Supplementary Fig. S21E). In susceptible species, TALEs (PthA5, PthA6, and PthA4) interact with the EBE regions of the *CsGRAS9* promoter, repressing *CsGRAS9* transcription and promoting susceptibility. In resistant species, where the EBE regions of the *CsGRAS9* promoter are absent or mutant, TALEs cannot recognize the *CsGRAS9* promoter, thus preventing transcriptional repression. *CsGRAS9* enhances resistance to citrus canker and regulates the expression of GA-related genes.

Building on these results, we present evidence that *GRAS9* expression plays a crucial role in controlling canker resistance. In citrus, natural variations and gene-edited mutations in the EBE regions of the *GRAS9* promoters disrupt the EBE*proGRAS9*-TALE complex, leading to transcriptional activation of *GRAS9* and enhanced canker resistance. The successful application of the PTG/Cas9 system, combined with the identification of promoter variations, provides a valuable genetic resource and a reliable platform for future molecular breeding efforts aimed at improving citrus resistance.

## Methods

### Plant materials and bacterial strains

Citrus plants, including wild-type Hong Kong kumquat (*Fortunella hindsii*), grapefruit (*Cirus paradisi* L.), ‘Anliu’ sweet orange (*C. sinensis*), and *proCsGRAS9*-edited seedlings, were cultivated in a greenhouse under controlled conditions of 28°C, 60% relative humidity (RH), and a 16:8 h light/dark photoperiod. *Xanthomonas citri* strains were grown on nutrient agar (NA) plates or in nutrient broth (NB) medium at 28°C, while *E*. *coli* strains were cultured in Luria-Bertani (LB) medium at 37°C. Appropriate antibiotics were added to bacterial growth media, including kanamycin (50 μg/ml), spectinomycin (100 μg/ml), and ampicillin (100 μg/ml). Strains and plasmids used in this study are listed in Supplementary Table S1.

### Sequencing of *tal* genes

Following the manufacturer's protocol and previous studies, the EZ-Tn5™ < KAN-2 > Insertion Kit was used to generate mutants by inserting the Tn5 transposon into the repeat regions of the *pthA5* and *pthA6* genes. (Haq et al. 2020). Mutants were selected using *Sph*I and *BamH*I restriction enzymes, and the complete sequences of *pthA5* and *pthA6 *were confirmed by sequencing with primer pairs TALE-SF/SR and Tn5-NF/NR. The primers used are listed in Supplementary Table S4.

### Phylogenetic tree construction and host target prediction

We obtained 77 complete genome sequences of *X*. *citri* strains from the NCBI database. AnnoTALE v1.4.1, was used to predict TALEs in each *X*. *citri* strain and to acquire the RVD sequence of TALEs (Grau et al. 2016). TALEs were grouped into classes based on RVDs that showed possible functional and evolutionary relationships (Grau et al. 2016). A disTAL module of the QueTAL suite was used to align and classify all *X. citri* strain TALEs based on their tandem repeat region sequences (Pérez-Quintero et al. 2015). FuncTAL was used to phylogenetically classify TALEs based on their DNA-binding specificities (Pérez-Quintero et al. 2015). Phylogenetic trees were constructed using the iTOL web server v5 (Letunic et al. 2021).

### Design and assembly of dGRAS9

Artificial dTALE (dGRAS9) was constructed by TALEN targeter 2.0 (https://tale-nt.cac.cornell.edu/node/add/talen) as previously described (Haq et al. 2020, Xu et al. 2024). The repeat regions of dGRAS9 targeted the 17-bp promoter region (AACTTCTTAAGTTTGAG) of the citrus gene *CsGRAS9* (Cs2g22130). *dGRAS9* was cloned into the plasmid pUC57 with the *Sph*I site, replaced with the *Sph*I fragment of *pthXo1* to generate pZY-*dGRAS9*, and then fused with pHM1 at the *Hind*III site to construct pHZY-*dGRAS9* (Table S1). Primers used in this study are listed in Supplementary Table S4.

### Expression Analysis of TALEs by Western Blotting

The strain Xcc049E was used to assay the function of the *tale* genes and *dGRAS9* in citrus canker formation (Li et al. 2014).Empty vector pHZY, along with plasmids pHZY-*pthA5*, pHZY-*pthA6*, pHZY-*pthA4*, and pHZY-*dGRAS9*, were introduced into Xcc049E cells via electroporation (2.5 kV, 4.5 ms). The expression of constructed plasmids was confirmed by Western blotting using anti-FLAG antibodies. Primers used in these experiments are listed in Supplementary Table S4.

### Pathogenicity and bacterial growth assays

For leaf inoculation, *Xcc* bacterial suspensions were diluted with double-distilled water and administered to citrus leaves using 1-mL needleless syringes (Fu et al. 2020). In addition, healthy citrus leaves were punctured with a 0.6-mm pin, followed by application of 6 μL of *Xcc* cultures to each puncture. The severity of *Xcc* infection was evaluated at 8 days post-inoculation (dpi), and canker lesion development was photographed at 8, 12, 16, 20, 21, and 27 dpi. All experiments were conducted in triplicate with three biological replicates.

Bacterial growth in the leaves was quantified by serial dilution of bacterial suspensions extracted from three leaf discs with visible lesions. The discs were homogenized in tubes containing 1 mL of double-distilled water and steel balls, and the bacterial suspensions were plated on nutrient-rich media with the appropriate antibiotic. Each treatment was performed in triplicate at different time points (Conforte et al. 2019).

### Microscopic analysis

*Xcc*-infected grapefruit leaves were cut into 4 mm × 4 mm square pieces using a sharp stainless-steel scalpel and immediately placed into fixative solution (FAA) at 4°C overnight. The samples were rinsed, dehydrated, and resin-infiltrated following the protocol outlined by Long et al (Long et al. 2021). Blocked samples were manually trimmed, stained with toluidine blue, and observed under a light microscope. Leaf thickness and cell size were quantified using CaseViewer software.

### TALgetter predictions in citrus plants and promoter sequence analysis

The promoter sequences of citrus genes, including a 2000 bp fragment upstream from the translation start site (ATG) and the amino acid sequences of CsGRAS9 homologs, were retrieved from the Citrus Pan-genome to Breeding Database (CPBD) (http://citrus.hzau.edu.cn). Binding sites recognized by TALEs PthA1, PthA4, PthA5, and PthA6 were predicted in the *CsGRAS9* promoter using the Target Finder tool (https://tale-nt.cac.cornell.edu/node/add/talef-off) and AnnoTALE (http://jstacs.de/index.php/PrediTALE). The Alphafold2 platform was used to predict the 3D structure of the *Xanthomonas* PthA5 protein using deep neural networks, and protein-DNA complexes were modeled using DP-DOCK and visualized in PyMol (http://www.pymol.org/pymol) (Gao et al. 2009). Cis-acting elements in the promoter regions of *GRAS9* homologs were identified using PlantCARE (http://bioinformatics.psb.ugent.be/webtools/plantcare/html/) (Lescot et al. 2002). Phylogenetic trees of *CsGRAS9* homologs were constructed using the MEGA X program after aligning the promoter sequences.

### RNA extraction and Quantitative real-time PCR

Citrus leaves were collected at 1, 2, and 7 dpi. Total RNA was extracted from both infected and uninfected citrus leaves, and first-strand cDNA synthesis was performed using the FastKing RT Kit with gDNase (TIANGEN, China). qRT-PCR was carried out on an ABI 7500 real-time system using the TransStart Top Green qPCR SuperMix (TRANS, China). The relative expression levels were normalized to *CsEf1a* as an internal control, and the comparative 2^−ΔΔCt^ method was used to determine fold changes in gene expression. The RT-qPCR primers are listed in Supplemental Table S4.

### Electrophoretic mobility shift assay

A 35-bp promoter fragment corresponding to the predicted TALE binding region upstream of *CsGRAS9*, *CmGRAS9*, and *MpGRAS9* was synthesized and labeled with Cy5. *BamH*I fragments of *pthA5*, *pthA6*, and *pthA4* were cloned into pET28a vector (containing a His tag). Recombinant PthA5-His, PthA6-His, and PthA4-His proteins were expressed in *E*. *coli* BL21 strains induced with 0.5 mM IPTG at 16°C overnight. After cell lysis, the supernatant was passed through a Ni-NTA Sefinose Resin column (BBI, C600791, USA). Various concentrations of unlabeled *CsGRAS9*, *CmGRAS9*, and *MpGRAS9* probes were used for competitive binding assays. For the EMSA, Cy5-labeled probes were incubated with PthA5-His, PthA6-His, or PthA4-His proteins, EMSA Binding Buffer, and poly dI-dC at 25°C for 25 minutes. The DNA-protein mixtures were resolved on 10% native polyacrylamide gel at 4°C for 1.5 hours at 100V in the dark (Xu et al. 2024). The fluorescent Cy5-labeled DNA was visualized using the Amersham Typhoon RGB imager (GE, Sweden).

### Yeast one-hybrid assay

The *CsGRAS9* and *FhGRAS9* were cloned into the p*LacZ* vector (bait). Multiple cloning sites (MCS) with *Xba*I, *EcoR*I, *BamH*I, *Xho*I, and *Pst*I restriction sites was inserted into the pBluescriptII SK(−) vector, which was digested with *Xba*I *and Pst*I. *BamH*I fragments of *pthA5* and *pthA6* were incorporated into the pB vector containing *EcoR*I and *BamH*I sites. The *EcoR*I-*BamH*I-cut fragments of *pthA5* and *pthA6* were then recombined into the pB42AD vector, producing pB42AD-PthA5 and pB42AD-PthA6 (prey). Yeast strain EGY48 was transformed with the bait and prey plasmids, grown on synthetic dropout (SD) medium lacking tryptophan (Trp) and uracil (Ura), and assayed on SD/-Trp-Ura plates with X-gal. Negative controls included yeast cells transformed with empty p*LacZ* or pB42AD vectors. Primers are listed in Supplementary Table S4.

### GUS activity assays

*BamH*I fragments of *pthA5*, *pthA6*, and *pthA4* replaced *avrXa10* in the pHB-*avrXa10 *vector through *BamH*I sites, serving as activators for transient expression in *N. benthamiana*. Reporter constructs (*pCsGRAS9*::GUS, *pCsGRAS9*-P1::GUS, *and pFhGRAS9*-P2::GUS) were generated by cloning *GRAS9 *homolog promoters into the pCAMBIA1381::GUS vector digested with *BamH*I and *Pst*I. The activator and reporter constructs were transformed into *Agrobacterium tumefaciens* strain GV3101 and grown on YEB medium containing kanamycin and rifampicin. Agroinfiltration was performed on *N. benthamiana* leaves following standard protocols (Xu et al. 2024). Leaf discs (10 mm in diameter) were collected at 2–3 dpi, stained with GUS buffer at 37°C overnight, and washed in graded ethanol to remove chlorophyll. GUS activity was quantified using 4-methylumbelliferyl-β-glucuronide. Primers are listed in Supplementary Table S4.

### Dual-luciferase reporter (dual-LUC) assay

The *pCsGRAS9*::LUC reporter was constructed by inserting the *CsGRAS9* promoter into the pGreenII0800-LUC vector, digested with *Hind*III and *BamH*I. The constructs were transformed into *A*. *tumefaciens* strain GV3101 and agroinfiltrated into *N. benthamiana* for dual-LUC assays (Long et al. 2021). Firefly (LUC) and Renilla (REN) luminescence ratios were determined using the Dual-Luciferase Reporter Gene Assay Kit (Shanghai, China) and the Dual-Glo® Luciferase Assay System (Promega). Fluorescence was observed using an imaging system. Primers are listed in Supplementary Table S4.

### Identification of GRAS9 proteins in citrus and other species

Citrus GRAS protein sequences were obtained from the Citrus Pan-genome to Breeding Database (CPBD) (http://citrus.hzau.edu.cn) and the NCBI database. Multi-sequence alignments of citrus GRAS proteins and conserved GRAS9 members were performed using MEGA 7.0, and phylogenetic trees were constructed from full-length amino acid sequences. The MEME suite (version 5.5.5) was employed to identify 20 conserved motifs in citrus GRAS9 proteins and predict protein functions (Dutta et al. 2021). Visualization was performed using TBtools software.

### Transcriptional activation activity assay

The coding sequences (CDS) of *CsLOB1* (positive control) and *CsGRAS9* were inserted into pGBKT7 to fuse with the GAL4 DNA-binding domain. Yeast strain AH109 was co-transformed with these vectors. Positive clones were selected on SD/-Leu/-Trp medium and assayed for transcriptional activation on SD/-Leu/-Trp/-His medium with or without X-α-gal (Long et al. 2021). Primers are listed in Supplementary Table S4.

### Subcellular Localization

The full-length CDS of *CsLOB1* and *CsGRAS9* were cloned into the pHB vector to generate YFP fusion constructs. pHB-CsLOB1 was used as a positive control, while the empty pHB vector served as a negative control. The vectors were transformed into *A. tumefaciens* strain EHA105, which was then infiltrated into *N*. *benthamiana* leaves. The nucleus was stained with DAPI (excitation at 405 nm), and YFP fluorescence signals (excitation at 514 nm, emission at 524–580 nm) were detected two days post-infiltration using a Leica TCS SPS-II confocal laser scanning microscope (Carl Zeiss SAS, Jena, Germany). Primers are listed in Supplementary Table S4.

### Generation of citrus mutants

The 23-bp gRNA sequence (target site: CCTTTCGCCTCTATATATATACA in the EBE region of *CsGRAS9* promoter) was designed using the online CRISPR-P2.0 design tool website (http://cbi.hzau.edu.cn/CRISPR2/). The gRNA-*CsGRAS9* fragment was formed by annealing and was immediately cloned into the AtU6-26-sgRNA-SK vector that was predigested with *Bsa*I. The *Spe*I fragment of the AtU6-26-*CsGRAS9*-sgRNA cassette was ligated into the pCAMBIA1300-p*YAO*-Cas9 vector containing Cas9 and GFP proteins. The construct was transferred into *A. tumefaciens* EHA105 and then transformed into ‘Anliu’ sweet orange epicotyls, as previously described (Tang et al. 2021a, b; Yan et al. 2015).

### Genotyping assay of CRISPR-edited mutations and Off-target analysis

Genomic DNA was extracted from *CsGRAS9* promoter-edited seedlings. Specific primers (Supplementary Table S4) were used to amplify the targetsites for mutation detection by Sanger sequencing. Off-target sites were screened using CRISPR-P2.0, and PCR products from potential off-target regions were sequenced for off-target analysis. Primers are listed in Supplementary Table S4.

### PPI and Yeast two-hybrid assays

The CsGRAS9 protein sequence was submitted to the STRING database (https://string-db.org/) to predict functional protein-protein interaction partners based on the *C*. *sinensis* genome (Szklarczyk et al. 2023). The interaction network was visualized using Cytoscape. The CDS of *CsGRAS9* was cloned into pGADT7 to create pGADT7-CsGRAS9, and *CsTAC1* was cloned into pGBKT7 to generate pGBKT7-CsTAC1. These vectors were co-transformed into yeast strain AH109, and transformed positive clones were selected on SD/-Leu/-Trp and SD/-Leu/-Trp/-His medium with or without X-*α*-gal to assess protein-protein interactions. Primers are listed in Supplementary Table S4.

## Supplementary Information

Supplementary Material 1.

Supplementary Material 2.

## Data Availability

The data will be available from the corresponding author upon reasonable request.

## Abbreviations

*Xcc*: *Xanthomonas citri* subsp. *citri*
CBC: Citrus bacterial canker
GRAS: Gibberellic acid insensitive (GAI), repressor of GA1–3 mutant (RGA), and scarecrow (SCR)
EMSA: Electrophoretic mobility shift assay
Y1H: Yeast one-hybrid
GUS: β-Glucuronidase
LUC: Luciferase
EBE: Effector binding element
TALE: Transcription activator-like effector
PTG/Cas9: Polycistronic tRNA–gRNA/Cas9
WT: Wild-type
GA: Gibberellin
TAC1: Telomerase activator1
HR: Hypersensitive response
T3SS: Type III secretion system
RVD: Repeat variable diresidue
CRRs: Central region of polymorphic repeats
CsLOB1: *Citrus sinensis* lateral organ boundary 1
InDel: Insertion/deletion
SNP: Single nucleotide polymorphism
*Xcm*: *Xanthomonas citri* pv. *malvacearum*
SCL: SCARECROW-LIKE
CPS: Ent-copalyl diphosphate synthase
KAO: Ent-kaurenoic acid oxidase
RGA: Repressor of ga1-3
LacZ: β-Galactosidase
As-1: Activation sequence-1
MEME: Multiple EM for Motif Elicitation
dTALE: Designer transcription activator-like effector
qRT-PCR: Quantitative Real Time PCR
*Xcv*: *Xanthomonas campestris* pv. *vesicatoria*
dsDNA: Double-stranded DNA
SA: Salicylic acid
JA: Jasmonic acid
ROS: Reactive oxygen species
*Xam*: *Xanthomonas axonopodis* pv. *manihotis*
BLB: Bacterial leaf blight
*Psa*: *Pseudomonas syringae* pv. *actinidiae*
GA3ox: GA 3-oxidase
GA20ox1: GA20 oxidase 1
GAMT2: GA methyltransferase 2
