# Supplementary material for: Two TAL effectors of Xanthomonas citri promote pustule formation by directly repressing the expression of GRAS transcription factor in citrus
Source: Mol Hortic. 2025 Mar 14;5:30. doi: 10.1186/s43897-024-00131-1 (PMC11907795; doi:10.1186/s43897-024-00131-1)
Supplement: Supplementary file 1 — Supplementary Material 1. [file 43897_2024_131_MOESM1_ESM.docx]

## Supplementary information

**
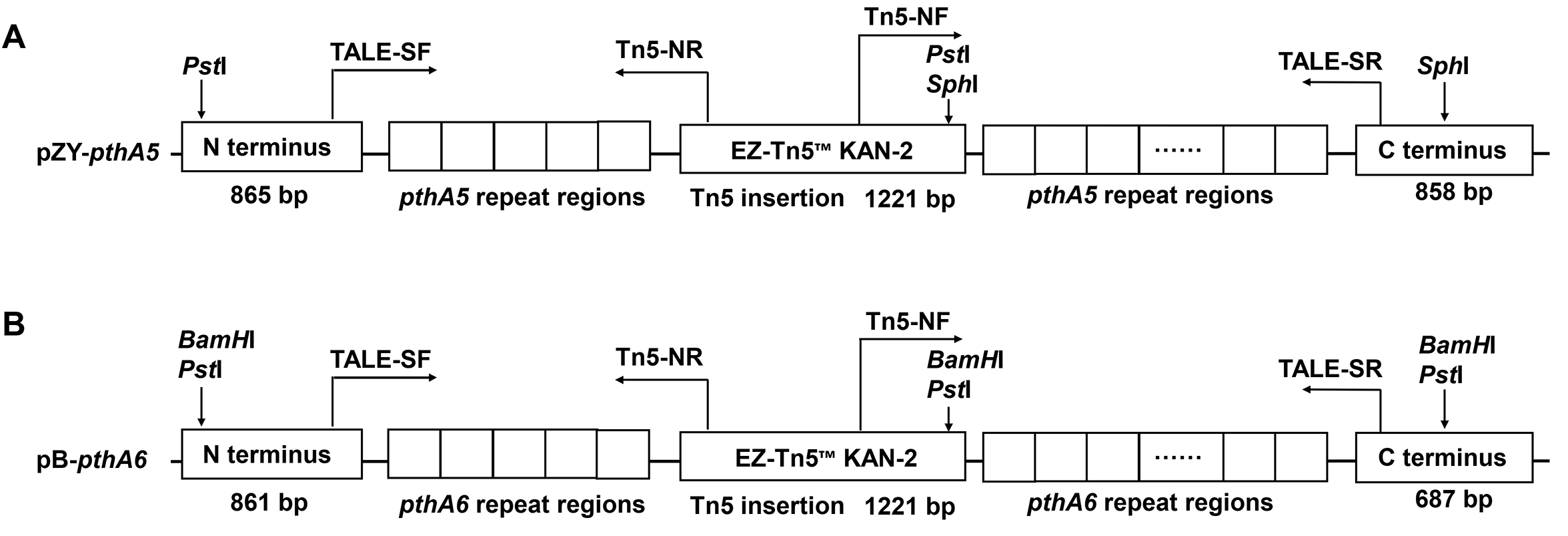
 Figure S1. Schematic diagram of the strategy used to sequence *pthA5* and *pthA6*.**

The EZ-Tn5™ < KAN-2 > Insertion Kit was used to construct several mutants in which the Tn5 transposon was inserted into the repeat regions of the *tal* gene in the pZY-*pthA5* (A) and pB-*pthA6* (B) recombinant plasmids. Single colonies were detected by restriction enzyme digestion and sequencing using primer pairs TALE-SF/SR and Tn5-NF/NR.

**Figure S2. Phylogeny of *Xanthomonas citri* TALEs.**

TALEs were obtained from Xcc003, Xcc086, and 78 complete genomes of *X*. *citri* strains by AnnoTALE. **(A)** RVD-based phylogenetic tree analysis of TALEs built by DisTAL. Their corresponding RVD repeats are shown together with phylogenetic tree. **(B)** Target affinity-based phylogenetic tree analysis of TALEs built by FuncTAL to investigate functional similarity. Their corresponding RVD repeats are shown together with phylogenetic tree. The clades of the neighbor-joining phylogenetic tree that contain PthA5 and PthA6 are shown together. PthA5 is highlighted in blue. PthA6 is highlighted in orange. **(C)** Geographic distribution of the sample sites of *Xcc* strains shown in (A) and (B) with different colors.


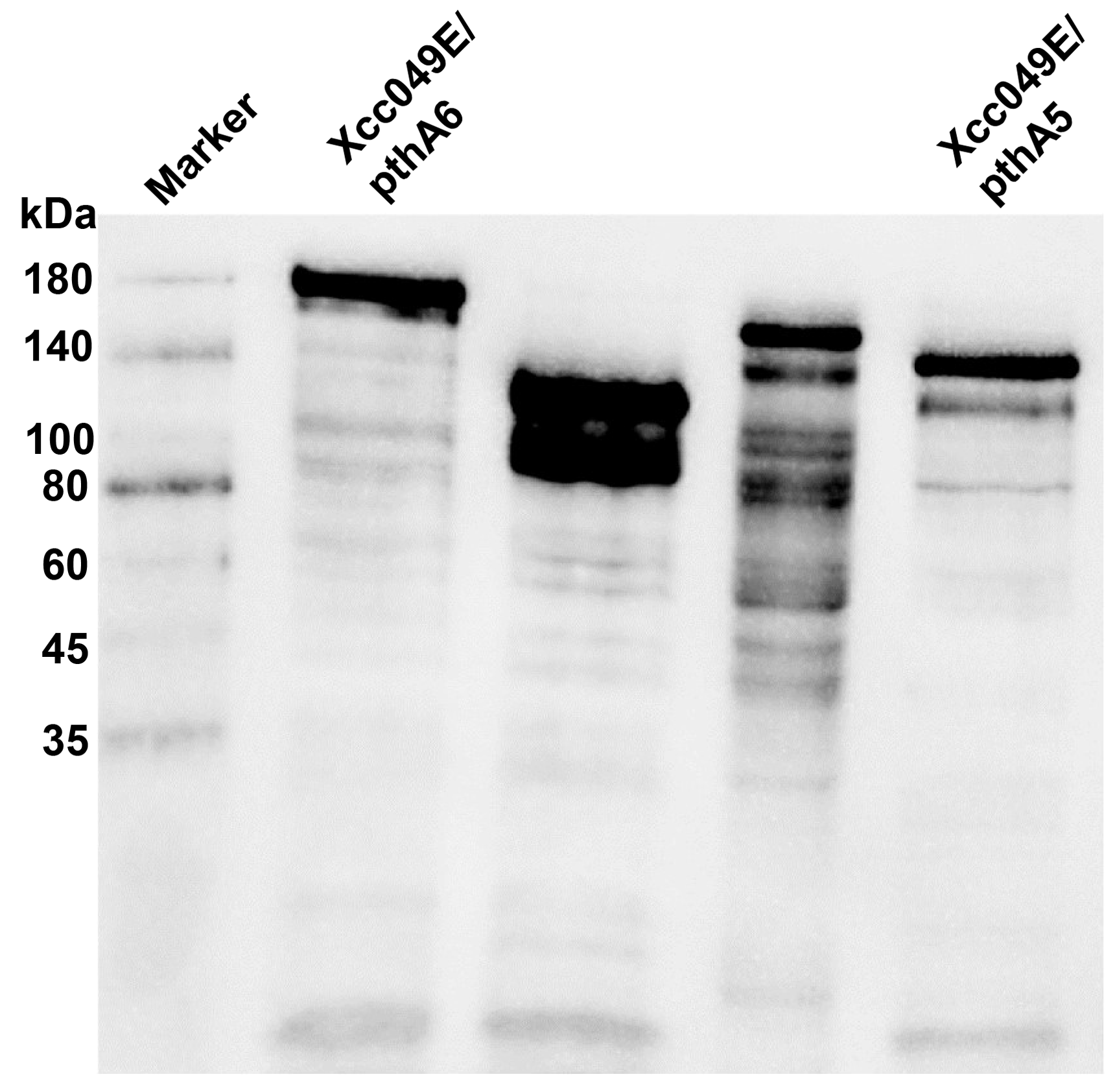


**Figure S3. Western blot analysis of PthA5 and PthA6 production in Xcc049E cells.**

The recombinant plasmids pHZY-*pthA6* and pHZY-*pthA5* were transferred into Xcc049E by electroporation. The productions of PthA5 and PthA6 were investigated by western blotting with an anti-FLAG primary antibody.


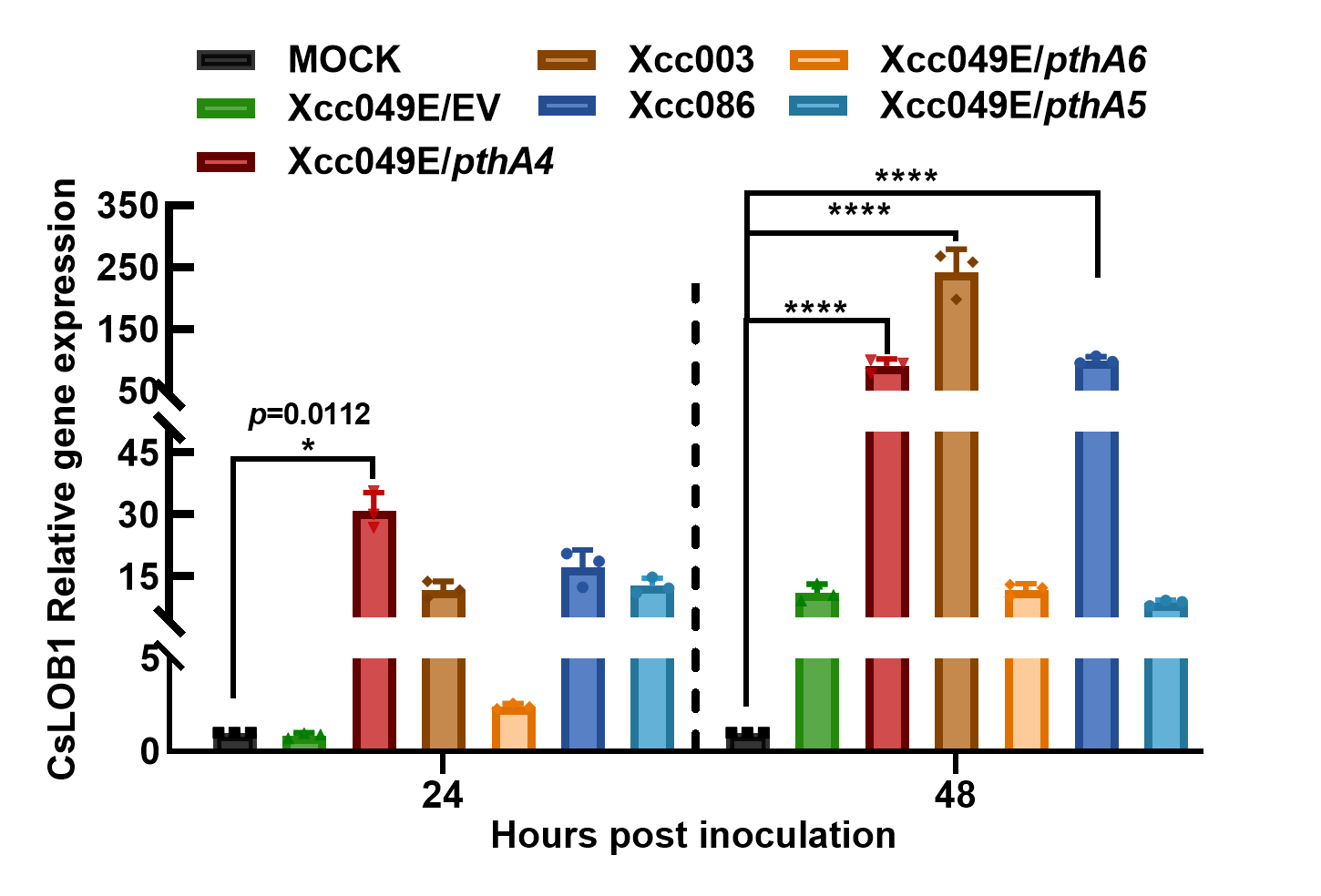


**Figure S4. *CsLOB1* expression in grapefruit leaves after inoculation with *Xcc* strains.**

*CsLOB1* expression was assessed by qRT-PCR, with *CsEf1a* as the normalization control. Three biological replicates were presented the mean ± SEM values. Asterisks indicate statistically significant differences between bacterial-inoculated leaves by two-way ANOVA with Dunnett's test: **p* < 0.05, *****p* < 0.0001.


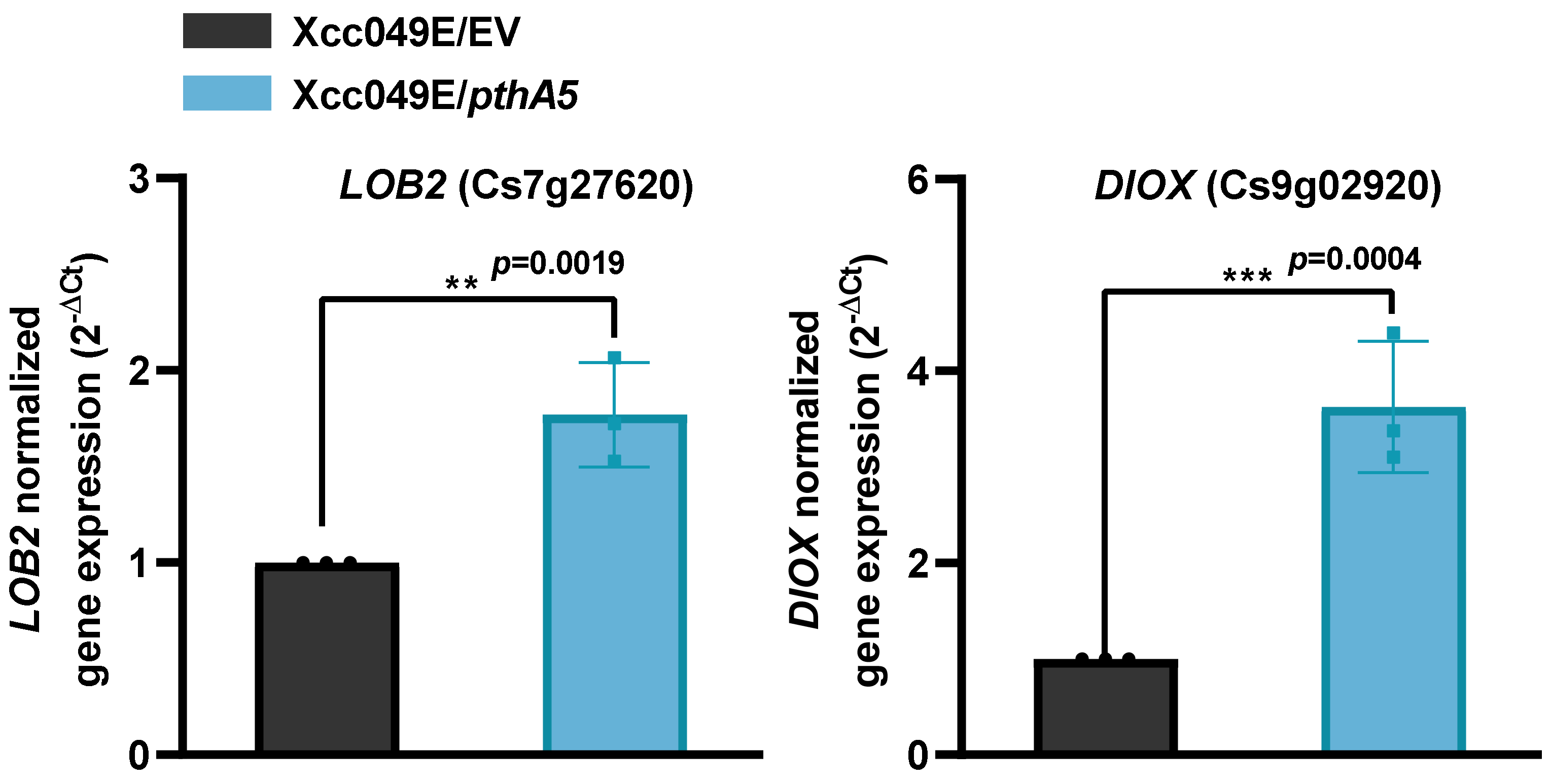


**Figure S5 *CsLOB2* and *CsDIOX* were induced in response to PthA5.**

*CsLOB2* and *CsDIOX* expression was assessed in Xcc049E/*pthA5* using qRT-PCR, with *CsEf1a* as the normalization control. Three biological replicates were presented the mean ± SEM values. Asterisks indicate statistically significant differences with Student’s *t* test (***p* < 0.01 and ****p* < 0.001) between bacterial-inoculated leaves.

**Figure S6. PthA5- and PthA1-binding elements in the *CsDIOX* promoter.**

Predicted EBE-binding site positions within the region upstream of the ATG (green) for *CsDIOX* promoter. EBE-binding sites are highlighted in yellow.


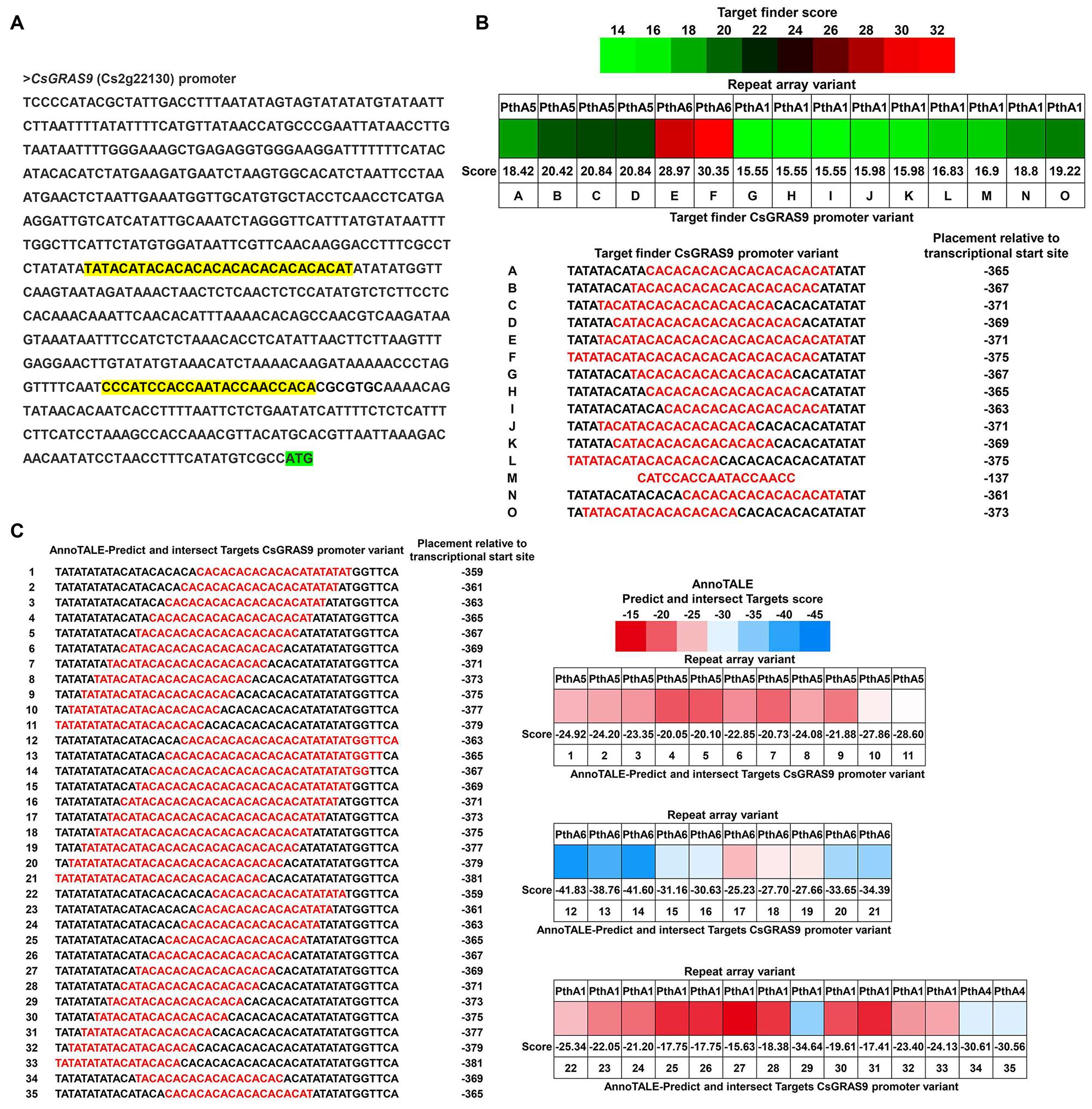


**Figure S7. PthA5- and PthA6-binding elements in the *CsGRAS9* promoter.**

(A) Predicted EBE-binding site positions within the region upstream of the ATG for *the CsGRAS9* promoter. EBE-binding sites are highlighted in yellow. (B) TAL Effector Nucleotide Targeter 2.0 predicted EBEs for TALEs (PthA1, PthA4, PthA5 and PthA6) on the *CsGRAS9* promoter by Target Finder tool. Scores are displayed in the boxes down the TALE names and in the form of a colored heat map, against Target Finder tool prediction scores (lower scores correspond to higher predicted binding affinity). (C) AnnoTALE predicted EBEs for TALEs (PthA1, PthA4, PthA5 and PthA6) on the *CsGRAS9* promoter by Predict and intersect Targets tool. Scores are displayed in the boxes down the TALE names and in the form of colored heat maps, against Predict and intersect Targets tool prediction scores (higher scores correspond to higher predicted binding affinity). Sequences are provided in Table S5-8.


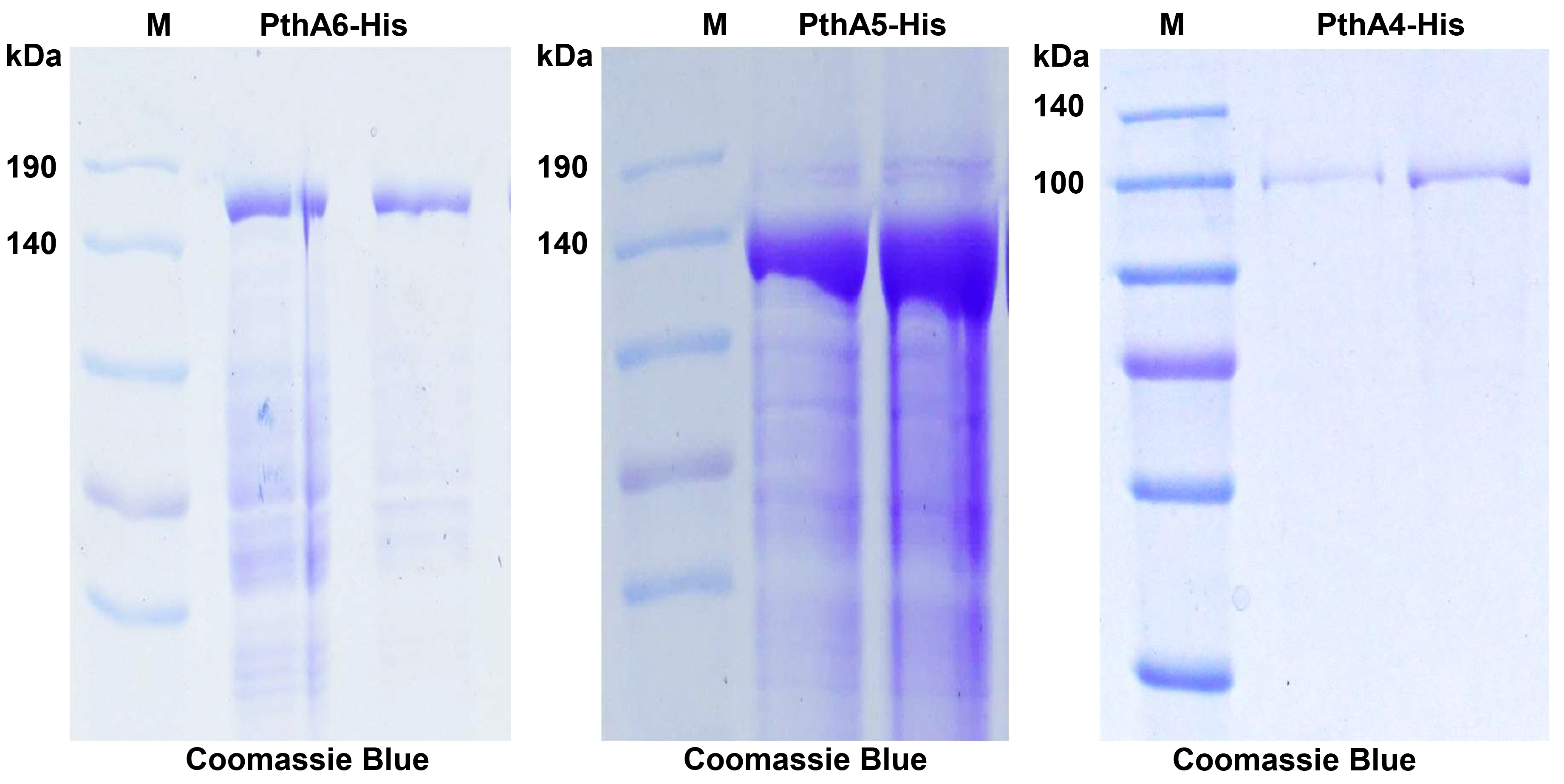


**Figure S8. His-tagged fusion proteins PthA6-His, PthA5-His, and PthA4-His were overexpressed in BL21 strains and purified.**

**
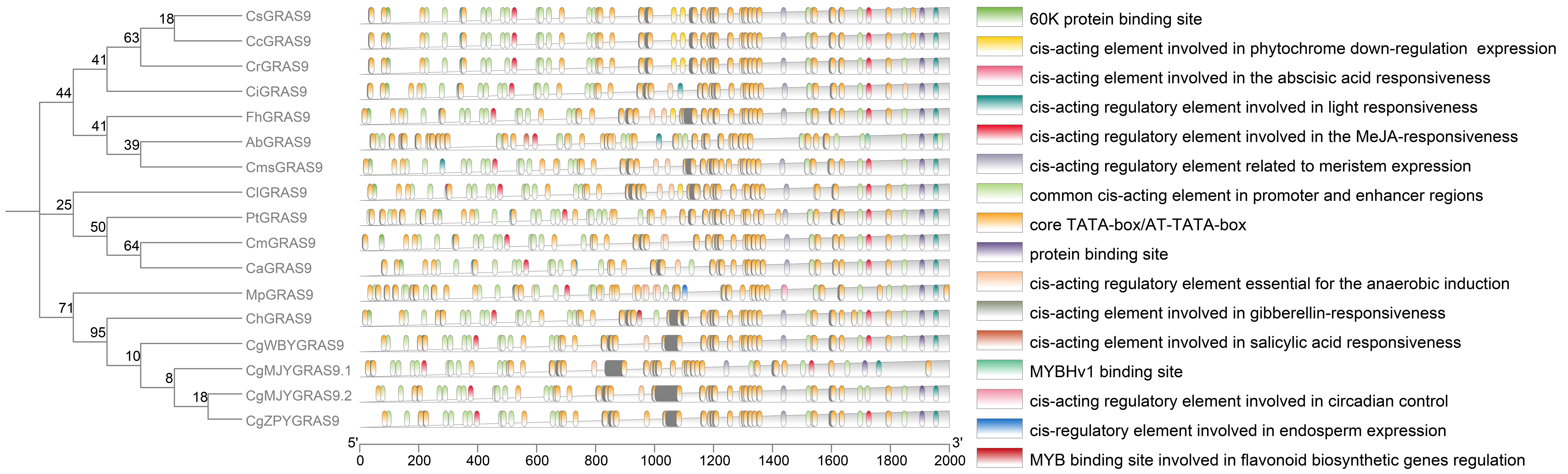
**

**Figure S9. Presence and frequency of cis-acting elements in different *GRAS9s* promoters in citrus plants.**

Analysis of cis-acting elements in *GRAS9s* across citrus plants.

**Figure S10. Promoter sequence** **comparison of *FhGRAS9* and *CsGRAS9*.**

(A) Bacterial growth in leaves of *F. hindsii* (Shan Jin Gan) after inoculation with *Xcc* by the infiltration method at 0, 1, 3, 5, 7, and 12 days post inoculation (dpi). Error bars represent the standard deviation of three biological replicates. Asterisks indicate statistically significant differences by two-way ANOVA with Tukey's test: (**p* < 0.05, ***p* < 0.01, ****p* < 0.001, *****p* < 0.0001) between the bacterial-inoculated leaves. **(B)** The alignment was generated by DNAMAN software. The conserved nucleotide sequences are highlighted in red, indicating homology levels 100%. **(C)** The promoter sequences of *FhGRAS9* in *F. hindsii* (Shan Jin Gan) were cloned. Sanger sequencing confirmation of the *FhGRAS9* promoter. EBE-binding sites are highlighted in red.

**
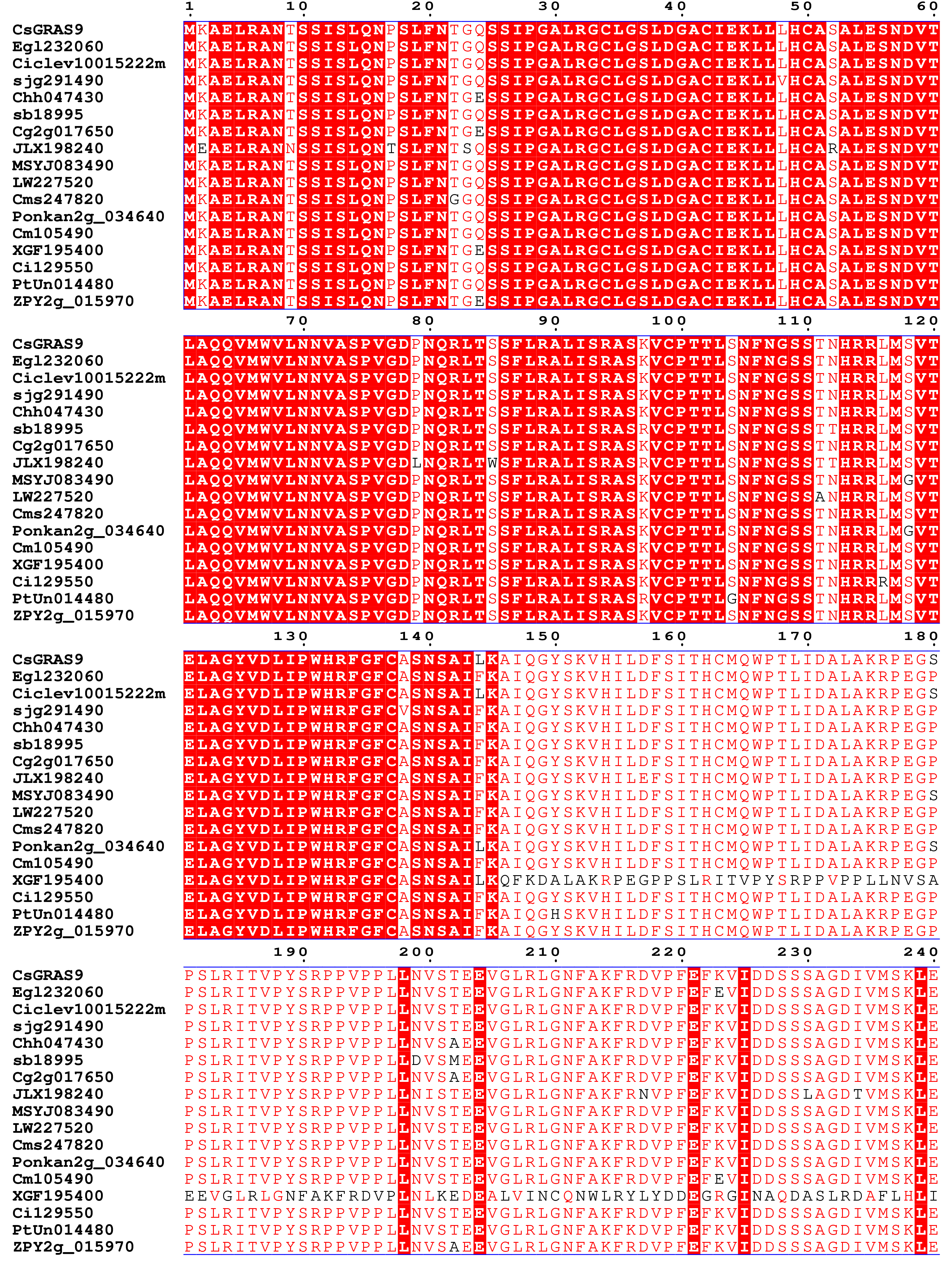
**

**Figure S11. Protein sequence comparison of GRAS9s in citrus.**

The alignment was generated using DNAMAN software. The conserved amino acids are highlighted in red. Black indicates different amino acids. Genes were listed in Table S3.


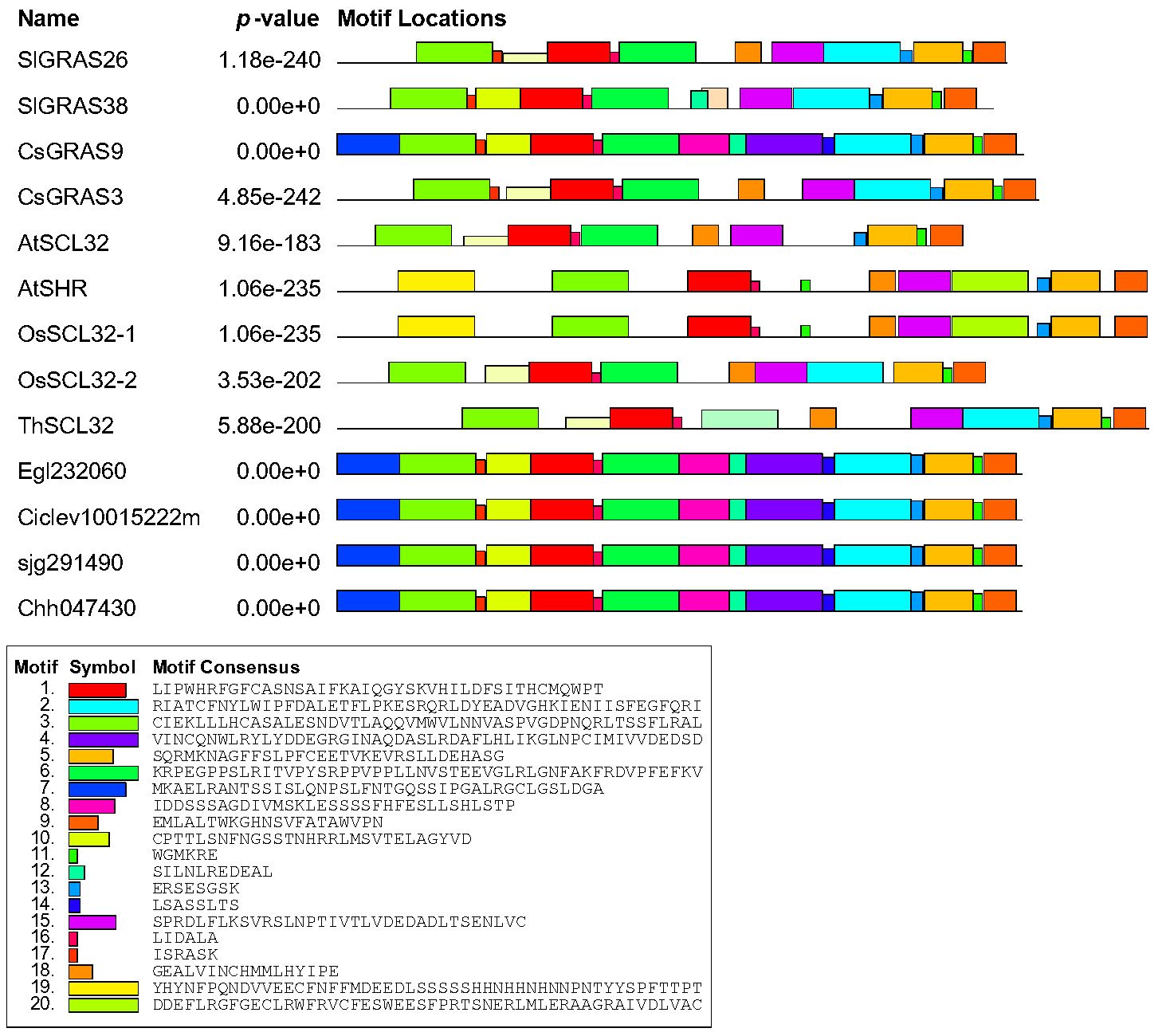


**Figure S12. MEME (Multiple EM for Motif Elicitation) analysis of the conserved motifs of GRAS9 homologs.**

The Motif symbol of GRAS9 proteins of *Citrus spp*., rice (*Oryza sativa*), *Arabidopsis thaliana*, *Tamarix hispida*, and tomato (*Solanum lycopersicum*) using MEME website ( <http://meme.nbcr.net/meme/website/meme-download.html> ).

**
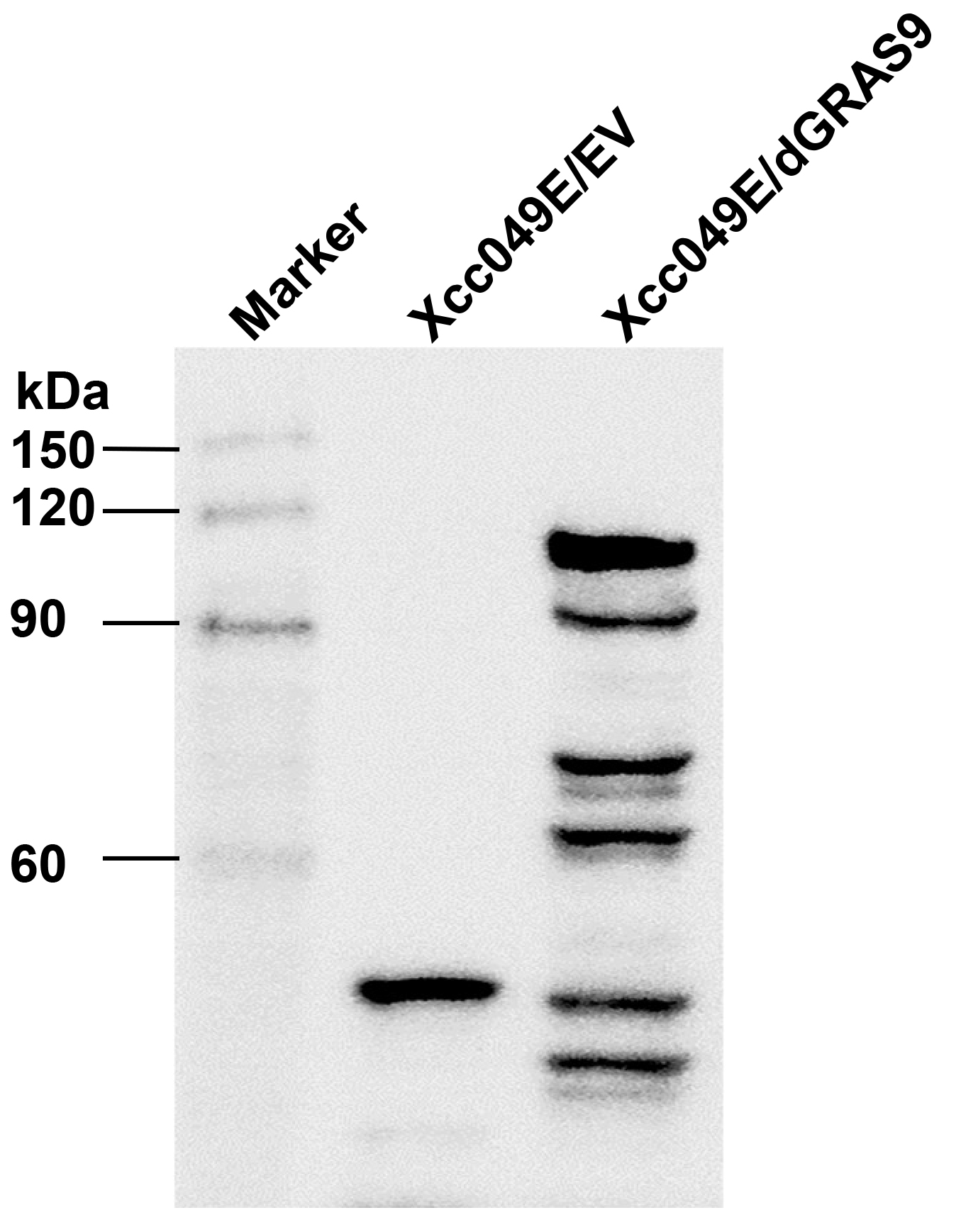
**

**Figure S13. Western blot analysis of dGRAS9 production in Xcc049E.**

Plasmids pHZY and pHZY-*dGRAS9* were transferred into Xcc049E by electroporation. The production of dGRAS9 was investigated by western blotting with an anti-FLAG primary antibody.

**
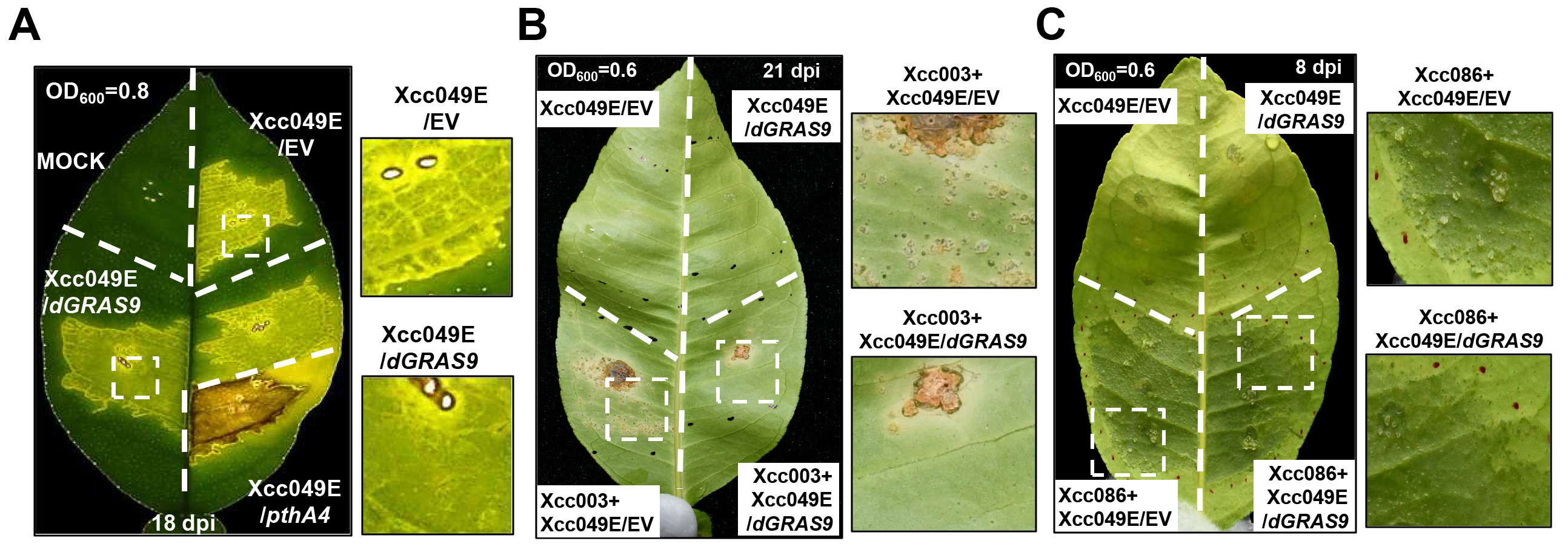
**

**Figure S14. *CsGRAS9* induces resistance in grapefruit.**

(A) Phenotypes of resistance to citrus canker induced by transiently expressed *CsGRAS9*. Leaves of grapefruit were infiltrated with *Xcc* suspensions (OD_600_=0.8) of Xcc049E/EV, Xcc049E/*dGRAS9*, Xcc049E/*pthA4* and water (Mock). Citrus canker symptoms were evaluated 18 days after bacterial inoculation. (B and C) Leaves of grapefruit were infiltrated with *Xcc* suspensions (OD_600_=0.6) of Xcc049E/EV, Xcc049E/*dGRAS9*, the mixed suspensions of Xcc003+Xcc049E/EV, Xcc003+Xcc049E/*dGRAS9*, Xcc086+Xcc049E/EV, Xcc086+Xcc049E/*dGRAS9* cells at a 1:1 ratio. Grapefruit leaves were inoculated with Xcc049E/EV and Xcc049E/*dGRAS9* suspensions, and these were inoculated with Xcc003+Xcc049E/EV (B) and Xcc086+Xcc049E/EV (C) suspensions as negative and positive controls to determine the severity of citrus canker, respectively. Citrus canker symptoms were evaluated 21 and 8 days after bacterial inoculation, respectively.

**Figure S15. CRISPR/Cas9-induced mutation T0*proGRAS9* line #13 in ‘Anliu’ *Citrus sinensis*.**

（**A**）Mutations in the alleles of *the CsGRAS9* promoter are shown for T0*proGRAS9* line #13. Red highlights indicate the positions where the mutations occurred. -: deletion. +: insertion; X number indicates the number of sequenced colonies. Nucleotides in red indicate gRNA. The underlined area in black (CCT) indicates the PAM. (**B**) Canker symptoms in T0*proGRAS9* line #13 after inoculation with *Xcc*. Sweet orange leaves were inoculated by the infiltration method with *Xcc* of mixed suspensions (OD_600_=1.2) of Xcc049E/*pthA4* and Xcc049E/*pthA5* or Xcc049E/*pthA6* cells at a 1:1 ratio using needleless syringes. Xcc049E/EV and complementation of Xcc049E with pthA4 were used as negative and positive controls, respectively. Citrus canker symptoms were evaluated 21days after inoculation.


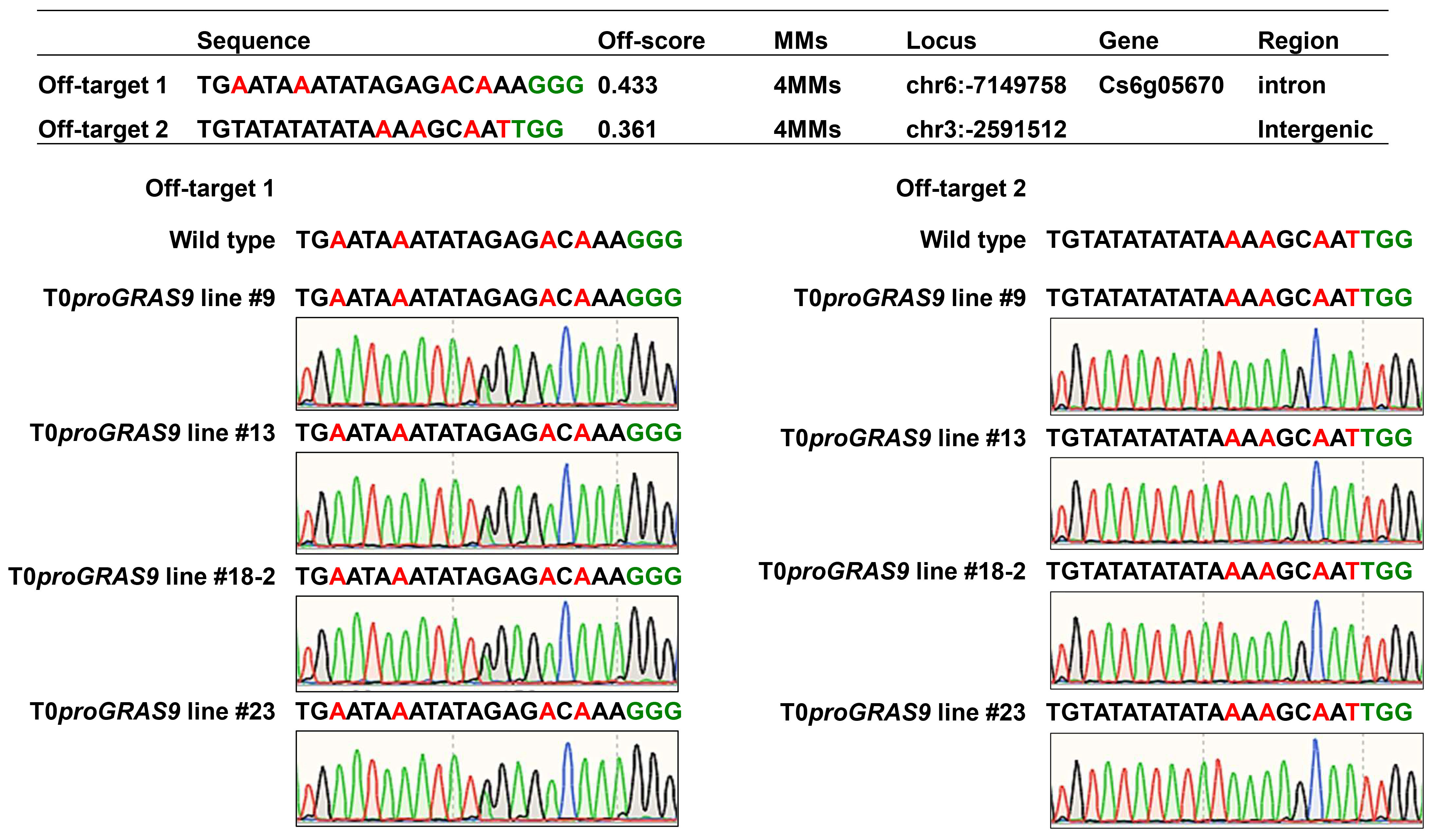


**Figure S16. Off-target analysis by PCR amplification and sequencing of leaves of *proCsGRAS9*-edited *C*. *sinensis* lines #9, #13, #18-2, and #23.**


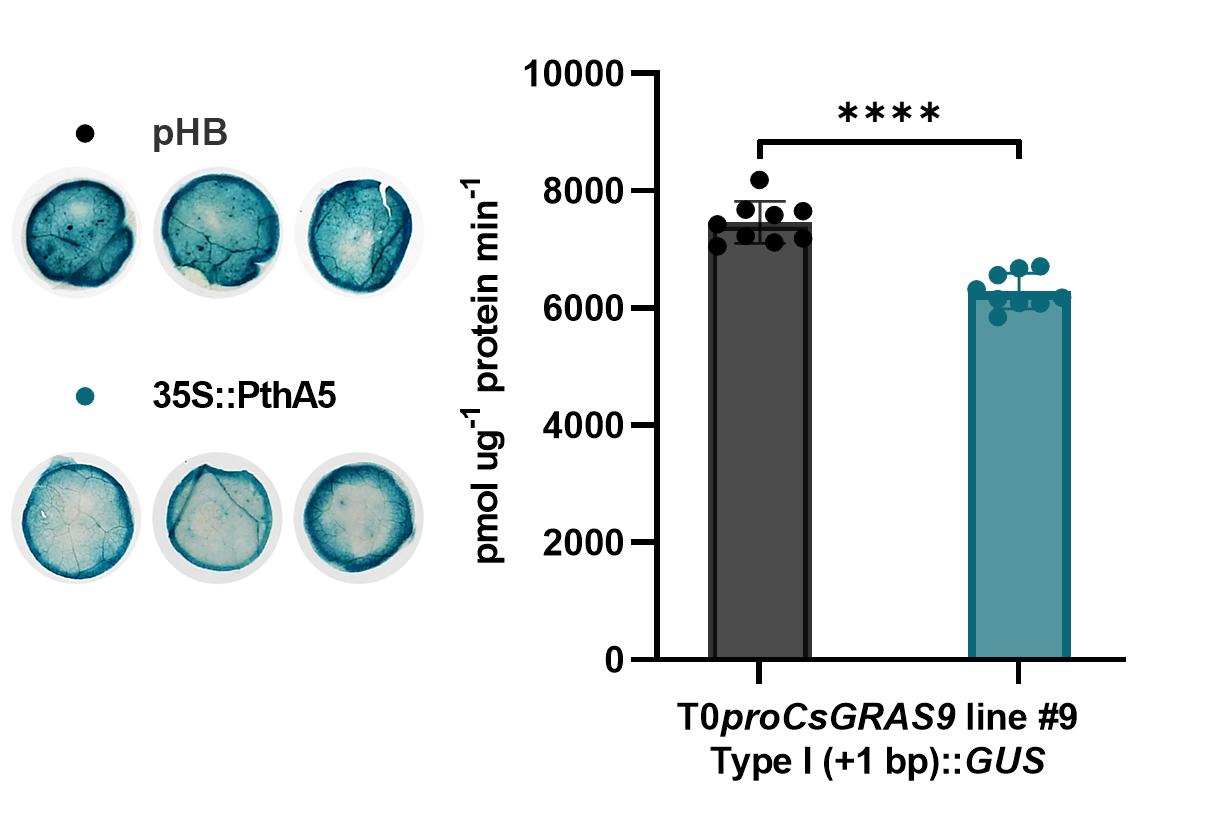


**Figure S17. Analysis of binding sites of PthA5 in the *CsGRAS9* promoter of T0*proGRAS9* line #9 ‘Anliu’ *Citrus sinensis*.**

The reporter vector T0*proGRAS9* line #9 Type I (+1bp)::*GUS* and truncated *GRAS9* promoter variants were constructed. The effector construct pHB-TALE086-36 and reporter vector T0*proGRAS9* line #9 Type I (+1bp)::*GUS* were used for transient expression in the *GUS* assays. *GUS* staining and activity assays were performed in *N*. *benthamiana* leaves with infiltration with the effector and reporter combinations as labeled constructs. Three biological replicates are presented as mean ± SEM values. Asterisks indicate statistically significant differences (*****p* < 0.0001, Student’s t-test).

**Figure S18. Canker symptoms in** **T0*proGRAS9* line #18-2 and WT after inoculation with *Xcc*.**

Leaves of WT (A) and T0*proGRAS9* line #18-2 (B) were inoculated by the infiltration method with *Xc*c of mixed suspensions (OD_600_=1.2) of Xcc049E/*pthA4* and Xcc049E/*pthA5* or Xcc049E/*pthA6* cells at a 1:1 ratio using needleless syringes. Xcc049E/EV and complementation of Xcc049E with *pthA4* were used as negative and positive controls, respectively. Citrus canker symptoms were evaluated 27 days after inoculation.

**Figure S19. Relative expression of *CsGRAS9* in the leaves of wild-type and *proCsGRAS9*-edited *C*. *sinensis* lines #9, #18-2, and #23 was measured at 48-hour post-inoculation (OD_600_=1.0).** *CsEf1a* was used as the constitutive standard. Four biological replicates are presented as the mean ± SEM values. Different letters indicate significant differences (*p* < 0.05), as analyzed by two-way ANOVA with Tukey's test.

**Figure S20. Interaction network of CsGRAS9 in citrus plants.** (**A**) Specific protein interactions between CsGRAS9 and other citrus proteins were determined using STRING. (**B**) Relative expression of *DOF4.6* and *CML30* in the leaves of WT grapefruit was measured 7-days post-inoculation (OD_600_=1.0). (**C**) Relative expression of *TAC1* in the leaves of WT grapefruit was measured 48-hour post-inoculation (OD_600_=1.0). *CsEf1a* was used as the constitutive standard. Three biological replicates are presented as mean ± SEM values. Asterisks indicate statistically significant differences (***p* < 0.01, ****p* < 0.001, Student’s t-test) between the bacterial-inoculated leaves. (**D**) Y2H assays showed that CsGRAS9 interacted with CsTAC1. An empty AD vector was used as the negative control.

**Figure S21.** **Molecular mechanism underlying TALEs regulating resistance to citrus canker through interactions with downstream gene *CsGRAS9* during *Xcc* infection. A-D** Relative expression levels of *GA20ox1* (*Cs1g09880*) (**A**), *GA3ox2* (*Cs4g20350*) (**B**), *GA3ox3* (*Cs4g17110*) (**C**), and *GAMT2* (*Cs9g03520*) (**D**) in grapefruit leaves were measured 48 hours post-inoculation with *Xcc049E*/*dGRAS9* (OD_600_ = 1.0), which induced the expression of *CsGRAS9*. *CsEf1a* was used as a constitutive standard. Three biological replicates are presented as the mean ± SEM values. Asterisks indicate statistically significant differences using Student’s t-test (***p* < 0.01, ****p* < 0.001, *****p* < 0.0001). **E** The proposed model of TALEs and *CsGRAS9* in modulating citrus canker resistance. The model contrasts susceptible (left) and resistant (right) citrus species. Arrows represent transcription activation, while flat lines indicate inhibitory.
